# Neutrophil Senescence Induced by Tumor-Derived HMGB1 Promotes Hepatocellular Carcinoma Progression through Neutrophil Extracellular Trap-Mediated Suppression of CD8^+^ T Cell Immunity

**DOI:** 10.34133/cancomm.0050

**Published:** 2026-09-11

**Authors:** Yaqian Duan, Shan Yu, Xuanyi Wang, Rong Wu, Yuqing Zhao, Xuehua Kong, Shiyu Cao, Dan Xiang, Rui Wu, Jingying Zhou, Yang Luo, Liang Duan

**Affiliations:** ^1^ Department of Laboratory Medicine, The Second Affiliated Hospital of Chongqing Medical University, Chongqing, P. R. China.; ^2^ Department of Pathology, The Second Affiliated Hospital of Chongqing Medical University, Chongqing, P. R. China.; ^3^ Department of Laboratory Medicine, The First Affiliated Hospital of Chongqing Medical University, Chongqing, P. R. China.; ^4^ School of Biomedical Sciences, The Chinese University of Hong Kong, Hong Kong SAR, P. R. China.; ^5^ Department of Laboratory Medicine, Chongqing General Hospital, School of Medicine, Chongqing University, Chongqing, P. R. China.

## Abstract

**Background:** Immune cells are essential components of the tumor microenvironment. Among them, neutrophils have gained increasing interest due to their marked heterogeneity. This study focused on aged neutrophils, aiming to uncover their mechanistic contribution to hepatocellular carcinoma (HCC) progression and evaluate their potential clinical importance. **Methods:** This study included analyses of 2 public datasets (GSE149614 and GSE189903) as well as a clinical cohort comprising 105 patients with HCC and 41 healthy controls. The abundance of aged neutrophils and CD8^+^ T cells as well as their clinical correlation were assessed, and their potential clinical value was explored. In vitro cellular experiments were used to dissect the molecular mechanisms responsible for CD8^+^ T cell suppression by aged neutrophils and the progression of neutrophil senescence. In addition, a variety of in vitro and in vivo experiments were performed to evaluate the involvement of aged neutrophils in HCC progression. **Results:** Peripheral blood and tumor tissues from HCC patients exhibited a marked accumulation of aged neutrophils, which was inversely correlated with CD8^+^ T cell abundance. Aged neutrophils released excessive neutrophil extracellular traps (NETs) via a caspase-3-dependent mechanism, thereby impairing CD8^+^ T cell proliferation and activation. Functionally, this suppression of CD8^+^ T cell activity promoted malignant behaviors of HCC cells, including enhanced proliferation, reduced apoptosis, and increased angiogenesis. In vivo, accumulation of aged neutrophils accelerated HCC progression through CD8^+^ T cell dysfunction, with minimal impact on distant metastasis. Mechanistically, HCC cell-derived high-mobility group box 1 (HMGB1) induced neutrophil senescence via activation of the Toll-like receptor 4 (TLR4) signaling pathway. Clinically, integrating circulating aged neutrophil with α-fetoprotein (AFP) substantially improved early HCC detection, while the CD8^+^ T cell-to-aged neutrophil ratio demonstrated strong potential for malignant risk stratification. **Conclusion:** These findings identify HCC cell-derived HMGB1 as a driver of neutrophil senescence and reveal that aged neutrophils promote HCC progression by inducing CD8^+^ T cell dysfunction through NET release. More importantly, aged neutrophils represent a robust noninvasive candidate marker for both HCC detection and assessment of malignant risk.

## Background

Hepatocellular carcinoma (HCC) constitutes the third major cause of cancer-related mortality on a global scale, with its high invasiveness and frequent recurrence leading to a poor prognosis [1]. Given that HCC is frequently diagnosed at advanced stages, there is an urgent need to improve the sensitivity and specificity of early detection and disease monitoring [2]. Although recent advances in surgical resection, locoregional therapies, and systemic treatments such as immune checkpoint inhibitors have improved clinical management, the overall survival remains unsatisfactory [3]. In addition to intrinsic tumor heterogeneity, the complex interactions among various cellular components in the tumor microenvironment (TME) profoundly influence HCC initiation, progression, and therapeutic responses [4]. Among these, the immunosuppressive state of immune cells is considered a key factor contributing to poor immunotherapy efficacy and sustained tumor progression in HCC. Therefore, elucidating the crosstalk among immune cells within the TME, particularly the mechanisms governing CD8^+^ T cell antitumor activity, is crucial for optimizing diagnostic and therapeutic strategies for HCC.

Neutrophils, traditionally regarded as innate immune effectors, have emerged as key players with their functional heterogeneity and central roles in tumor immunity [5,6]. Growing evidence indicates that neutrophils exhibit remarkable plasticity within the TME and adopt distinct functional states. For instance, immunosuppressive tumor-associated neutrophils secreted C–C motif chemokine ligand 4 to reprogram adjacent tumor cells toward a tumor-initiating phenotype, thereby evading CD8^+^ T cell-mediated cytotoxicity [7]. In addition, HCC-derived exosomes increased miR-362-5p levels in neutrophils, enhancing their survival, chemotaxis, and immunosuppressive phenotypes, which in turn promoted hepatic neutrophil infiltration and accelerated tumor progression [8]. Our previous research established that tumor-associated neutrophils could promote HCC growth, angiogenesis, and metastasis by releasing neutrophil extracellular traps (NETs) [9]. Although the use of immune checkpoint inhibitors has shown encouraging outcomes in advanced HCC, some obstacles remain, including limited response rates, primary or acquired resistance, and possible systemic toxicities, underscoring the critical need for innovative treatment strategies [3].

CD8^+^ T cells are the principal cytotoxic effectors in antitumor immunity, but within the TME, they frequently become dysfunctional, as evidenced by sustained up-regulation of inhibitory receptors, reduced cytotoxic capacity, and impaired cytokine production [10]. While tumor cell-intrinsic mechanisms have long been recognized as key drivers of CD8^+^ T cell dysfunction, emerging evidence highlights that immune cell interactions are equally critical in shaping T cell fate. For example, in HCC, tumor endothelial cell-derived glycoprotein nonmetastatic melanoma protein B induced dysfunction of tumor-infiltrating CD8^+^ T cells [11]. Notably, macrophages were shown to further aggravate CD8^+^ T cell dysfunction through metabolites such as itaconate, which enhanced succinate-dependent H3K4me3 modification of eomes, ultimately promoting tumor progression [12]. Dendritic cells contributed to immunosuppression and tumor immune evasion by producing indoleamine 2,3-dioxygenase, thereby suppressing CD8^+^ T cell expansion and effector responses [13]. Similarly, myeloid-derived suppressor cells suppressed CD8^+^ T cell-mediated antitumor immunity through elevated expression of arginase-1 and inducible nitric oxide synthase, thereby fostering an immunosuppressive TME [13,14]. These findings underscored that, beyond direct tumor-derived signals, the interplay among immune cells themselves played an essential role in driving CD8^+^ T cell dysfunction. Clarifying these complex cellular interactions is therefore crucial for unraveling mechanisms of immune evasion and for informing the development of more effective immunotherapeutic strategies.

Recent studies suggest that neutrophils are intimately involved in CD8^+^ T cell dysfunction. They can impair CD8^+^ T cell infiltration, suppress their activation and cytotoxicity, and promote T cell dysfunction, thereby fostering tumor progression [15]. Notably, a senescent neutrophil subset had been identified, characterized by prolonged lifespan, altered surface markers, and enhanced immunomodulatory potential [16,17]. Neutrophil senescence was regulated by circadian rhythms and exogenous stimuli [18]. For instance, microbiota-derived metabolites might translocate across the gut barrier, triggering senescence via Toll-like receptor (TLR)/myeloid differentiation primary response 88 signaling [19]. Although neutrophil senescence served homeostatic functions under physiological conditions, excessive senescence in pathological contexts could cause tissue damage and immune dysregulation [20]. The proinflammatory activity of neutrophils was positively correlated with their degree of senescence [19]. In breast cancer, aged neutrophils generated mitochondria-dependent vital NETs that promoted pulmonary metastasis by capturing circulating tumor cells [21]. In prostate cancer, the apolipoprotein E/triggering receptor expressed on myeloid cells 2/extracellular signal-regulated kinase axis induced immunosuppressive neutrophil senescence, with interleukin-6 (IL-6)/signal transducer and activator of transcription 3-driven secretome reprogramming supporting a protumoral phenotype [22]. Additionally, human immunodeficiency virus type 1 infection also elevated neutrophil aging, which suppressed CD8^+^ T cell-derived interferon-γ (IFN-γ) and tumor necrosis factor-α (TNF-α) more potently than non-aged neutrophils [23]. Despite these advances, the distribution, functional states, and impact of aged neutrophils on CD8^+^ T cell immunity in HCC remain undefined.

Despite advances in HCC immunotherapy, the mechanisms underlying CD8^+^ T cell dysfunction remain incompletely understood. The present study aimed to explore the immunosuppressive factors regulating CD8^+^ T cell function, with a focus on the role of aged neutrophils in the TME. To this end, we conducted a systematic analysis of clinical samples from HCC patients and mouse models, combined with in vitro functional experiments, to reveal the specific role and molecular mechanisms of aged neutrophils in the formation of the immunosuppressive microenvironment in HCC.

## Materials and Methods

### Patient specimens

A total of 105 HCC patients and 41 healthy controls were enrolled in this study. Among them, peripheral blood samples were collected from 100 HCC patients and 41 healthy controls; tumor tissues and paired adjacent normal tissue samples were collected from the other 5 HCC patients. All samples were acquired at the Second Affiliated Hospital of Chongqing Medical University from August 2024 to July 2025. The 100 HCC patients who provided peripheral blood samples met the following inclusion criteria: They were newly diagnosed and had not received any surgery, immunotherapy, or chemotherapy. Following flow cytometric (FCM) analysis, serum isolation was performed by centrifuging all peripheral blood samples at 2,000*g* for 10 min at room temperature. The resulting serum underwent cryopreservation at −80 °C pending further analysis. Additionally, tissue samples were collected from surgically treated patients who were newly diagnosed and had not undergone any prior surgery, immunotherapy, or chemotherapy. All samples were obtained and approved by the Institutional Ethics Committee of the Second Affiliated Hospital of Chongqing Medical University in agreement with the Declaration of Helsinki (no. 2024-067).

### Cell lines

The human normal liver cell line THLE-2 (CL-0833) and human HCC cell line HuH7 (CL-0120) were purchased from Procell Life Science & Technology Co. Ltd. The mouse HCC cell line Hepa 1-6 (iCell-m019) was purchased from Cellverse Co. Ltd. The HuH7 cell line stably expressing luciferase (HuH7-LUC) was established by transfecting HuH7 cells with recombinant lentivirus carrying the luciferase reporter gene (U24E03MF, Applied Biological Materials Inc.). The human hepatoblastoma cell line HepG2 and human umbilical vein endothelial cell line HUVEC were obtained from the Central Laboratory of the Second Affiliated Hospital, Chongqing Medical University [9]. All cell lines were cultured in Dulbecco’s modified Eagle’s medium (DMEM, Gibco) with 10% fetal bovine serum (FBS, Cellmax) and 1% penicillin/streptomycin (Beyotime) at 37 °C in an atmosphere of 5% CO_2_.

### Mice

Two- or 6-week-old male C57BL/6 and 6-week-old male BALB/c nude mice were obtained from Jiangsu Huachuang Sino Pharma Tech Co. Ltd. Mice were housed in ventilated cages with a maximum of 5 animals per cage under specific pathogen-free conditions. They were maintained on a 12-h light/12-h dark cycle, with ambient temperature and humidity controlled at 24 °C and 50%, respectively. All mice were monitored daily and euthanized in accordance with established humane endpoint criteria, which included a body weight loss of >20% or the presence of signs of distress such as lethargy or other indicators of suffering. Upon study completion, mice were euthanized using CO_2_ inhalation. All animal procedures were approved by the Animal Ethics Committee of the Second Affiliated Hospital of Chongqing Medical University (approval no. IACUC-SAHCQMU-2024-00053).

### Isolation and culture of human neutrophils and NET formation assay

Neutrophils were obtained from peripheral blood of 8 randomly selected healthy controls using a commercial human neutrophil isolation kit (P9040; Solarbio) and subsequently cultured in DMEM supplemented with 10% FBS. Freshly isolated neutrophils were stimulated with lipopolysaccharide (LPS; 10 ng/ml; L2880; Sigma) or granulocyte–macrophage colony-stimulating factor (GM-CSF; 100 ng/ml; 10015-HNAH; Sino Biological) to induce neutrophil senescence [23]. For the NET formation assay, neutrophils were treated with phorbol 12-myristate 13-acetate (PMA; 100 ng/ml; IP1010; Solarbio) for 4 h. NET purification was performed as previously described [9]. Purified NETs (2 μg/ml) were applied to CD8^+^ T cells for downstream analyses. NET-associated double-stranded DNA was enzymatically degraded using deoxyribonuclease I (DNase I; Sigma-Aldrich) for 4 h prior to subsequent assays.

### Isolation and culture of human CD8^+^ T cells

Peripheral blood mononuclear cells were isolated from 8 randomly selected healthy controls using a commercial human neutrophil isolation kit. CD8^+^ T cells were then purified from the peripheral blood mononuclear cells using Dynabeads (11348D; Invitrogen) and cultured in RPMI 1640 medium (Gibco) supplemented with 2 mM l-glutamine (Thermo Fisher), 1% Minimum Essential Medium nonessential amino acids (Procell), 1 mM sodium pyruvate (Procell), 10% FBS, and 1% penicillin/streptomycin. To promote activation and proliferation, CD8^+^ T cells were cultured with anti-CD3/CD28 dynabeads (11131D; Thermo Fisher) and recombinant IL-2 (30 U/ml; Thermo Fisher). Dynabeads were removed, and CD8^+^ T cells along with the conditioned medium (CM) were collected for subsequent analysis.

### Coculture assay

For CD8^+^ T cell analysis in 6-well plates, neutrophils (5 × 10^5^ cells/well)—either LPS- or GM-CSF-treated (representing aged neutrophils, AN) or untreated (representing neutrophils, Neu)—were cultured in the upper chamber of a 0.4-μm transwell system. Human CD8^+^ T cells (2 × 10^6^ cells/well)—either stimulated with anti-CD3/CD28 Dynabeads (representing activated CD8^+^ T cells, AT) or left unstimulated (representing CD8^+^ T)—were seeded in the lower chamber and cocultured for 12 h. Then, neutrophils were removed, and CD8^+^ T cells were cultured for an additional 48 h. For neutrophil analysis, THLE-2 (5 × 10^5^ cells/well), HepG2 (5 × 10^5^ cells/well), and HuH7 (5 × 10^5^ cells/well) were cultured in the upper chamber of a 0.4-μm transwell system, and neutrophils (2 × 10^6^ cells/well) were cultured in the lower chamber for 6 h.

### Cell proliferation assay

HepG2 (3 × 10^3^ cells/well) and HuH7 (3 × 10^3^ cells/well) were cultured and then treated with CM-CD8^+^ T, CM-AT (activated CD8^+^ T), CM-(AT + Neu), or CM-(AT + AN). Cell proliferation was determined with a Cell Counting Kit-8 (CCK-8) assay (MI00615, Mishushengwu). Optical density (OD) at 450 nm was measured once per day for 3 d using a microplate reader, and each experiment was repeated 3 times.

### Cell migration and invasion assay

HepG2 (2 × 10^4^ cells/well) and HuH7 (2 × 10^4^ cells/well) were cultured in the upper chamber of an 8-μm transwell system (coated with Matrigel for invasion assays) and incubated in serum-free DMEM, while CM-CD8^+^ T, CM-AT, CM-(AT + Neu), or CM-(AT + AN) with 20% FBS were added to the lower chamber. After 48 h of incubation, transmembrane cells were washed, fixed with 4% paraformaldehyde, subjected to crystal violet staining, and subsequently visualized by microscopy. Each experiment was repeated 3 times.

### Matrigel tube formation assay

HUVECs (1 × 10^4^ cells/well) were cultured on 96-well plates coated with Matrigel (0827045; Xiamen Mogengel) and incubated with CM-CD8^+^ T, CM-AT, CM-(AT + Neu), or CM-(AT + AN). Capillary-like tube formation was examined and counted under a microscope following 6 h of incubation, with each experiment independently repeated 3 times.

### Mouse liver cancer models

#### Diethylnitrosamine-induced spontaneous model

Two-week-old C57BL/6 mice received an intraperitoneal injection of diethylnitrosamine (25 mg/kg; HY-N7434; MedChemExpress), followed 4 weeks later by intraperitoneal injections of CCl₄ (1:4, v/v in olive oil; Macklin) at 2 ml/kg twice weekly for 14 weeks. During the subsequent 2 weeks, mice received daily oral gavage with phosphate-buffered saline (PBS), LPS (1 mg/kg), or an antibiotic cocktail (ABX) containing 4 broad-spectrum antibiotics: vancomycin (100 mg/kg), ampicillin (200 mg/kg), metronidazole (200 mg/kg) and streptomycin (200 mg/kg). For neutralization experiments, mice were intraperitoneally injected with anti-CD8α antibody (200 μg per mouse; BE0061; Bio X Cell) twice per week. Mice were euthanized at 22 weeks for subsequent analysis.

#### Hepa 1-6 cell-derived subcutaneous model

(a) Six-week-old C57BL/6 mice were administered daily oral gavage of LPS, PBS, or ABX and intraperitoneally injected with anti-CD8α antibody (200 μg per mouse) twice per week. After 1 week of pretreatment, Hepa 1-6 cells (2 × 10^6^) were subcutaneously implanted. Upon the development of palpable subcutaneous tumors, the same regimen of daily gavage and anti-CD8α antibody administration twice per week was resumed. (b) Six-week-old C57BL/6 mice were subcutaneously injected with Hepa 1-6 cells (2 × 10^6^). Upon the formation of palpable subcutaneous tumors, intratumoral injections of murine recombinant high-mobility group box 1 protein (Hmgb1; 10 μg per mouse; HY-P73104A, MedChemExpress), glycyrrhizic acid (Gly; 10 mg/kg; HY-N0184, MedChemExpress), or TAK-242 (1 mg/kg; HY-11109, MedChemExpress) were administered every 2 d. Tumor growth at the subcutaneous site was assessed every 2 d with vernier calipers, and volumes were calculated as 1/2 × (*R*_max_ × *R*_min_^2^), with *R* denoting the tumor diameter. Tumor tissues were harvested for further experimental analyses.

#### HuH7-LUC cell-derived lung metastasis model

Six-week-old BALB/c nude mice were injected via the tail vein with HuH7-LUC cells (2 × 10^6^) pretreated with different CM. For in vivo bioluminescence imaging (AniView100 Pro, Guangzhou Biolight Biotechnology), d-luciferin was injected intraperitoneally at 15 mg/kg. Lung tissues were harvested 30 d post-injection for further analyses.

### Immunohistochemistry staining

Paraffin-embedded tissue sections from 3 randomly selected mice in each group were first rehydrated and underwent antigen retrieval using microwave heating, followed by inhibition of endogenous peroxidase activity. Then, the sections were incubated with anti-Ki67 (1:300; GB111141; Servicebio), anti-CD31 (1:300; GB113151; Servicebio), and anti-vascular endothelial growth factor (VEGF; 1:300; AF5131; Affinity), overnight at 4 °C, followed by secondary antibody labeling with the peroxidase enzyme (1:500; AFIHC003; AiFang) for 50 min at room temperature. After 3,3′-diaminobenzidine staining, the sections were counterstained with hematoxylin and subsequently evaluated under an optical microscope (Olympus Bx53). Image quantification was conducted using Image-Pro Plus 6.0 software (Media Cybernetics Inc.).

### Immunofluorescence staining

For cell immunofluorescence (IF) staining, neutrophils (5 × 10^5^ cells/well) were exposed to the designated stimulant. After fixation with 4% paraformaldehyde, cells were permeabilized with 0.1% Triton X-100, followed by blocking with goat serum at 37 °C for 1 h. Subsequently, cells were incubated overnight at 4 °C with anti-myeloperoxidase (MPO; 1:500; 66177-1-Ig; Proteintech) and anti-citrullinated histone H3 (CitH3; 1:500; ab5103; Abcam) antibodies. Then, cells were incubated with fluorescence-conjugated secondary antibodies, followed by nuclear staining with 4′,6-diamidino-2-phenylindole (DAPI; AR1177; Boster) for 10 min at 37 °C. The slides were washed, mounted with antifade medium (AR1109; Boster), and imaged using a confocal microscope (FV12-IXCOV, Olympus). For tissue IF staining, tissue sections were pretreated following the same procedure as immunohistochemistry (IHC). Human liver sections from 5 HCC patients were incubated overnight at 4 °C with the following primary antibodies: anti-CD66b (1:2,000; ab300122; Abcam), anti-C–X–C chemokine receptor type 4 (CXCR4; 1:2,000; ab181020; Abcam), anti-CD4 (1:3,000; ab133616; Abcam), and anti-CD8 (1:3,000; ab237709; Abcam). Mouse tumor tissue sections from 3 randomly selected mice in each group were incubated overnight at 4 °C with anti-lymphocyte antigen 6 complex locus G (Ly6G; 1:2,000; ab238132; Abcam), anti-CXCR4 (1:2,000; ab124824; Abcam), anti-CD8 (1:3,000; 98941; Cell Signaling Technology), and anti-CitH3 (ab281584; Abcam). Then, sections were incubated with fluorescence-conjugated secondary antibodies, counterstained with DAPI for nuclei, and visualized using a fluorescence microscope (FV12-IXCOV, Olympus).

### Enzyme-linked immunosorbent assay

Enzyme-linked immunosorbent assay (ELISA) was performed following the manufacturer’s instructions. The following ELISA kits were used: human perforin (MM1156H1; Meimian), human granzyme B (Gzm B; MM-0434H1; Meimian), human IFN-γ (E-EL-H0108; Elabscience), human high-mobility group box 1 (HMGB1; E-EL-H1554; Elabscience), mouse perforin (E-EL-M0890; Elabscience), and mouse Gzm B (E-EL-M0594; Elabscience). The culture supernatant of MPO–DNA complexes was analyzed by ELISA using a cell death detection ELISA kit (11774425001; Roche). Briefly, 96-well plates were precoated with 5 μg/ml anti-MPO capture antibody (AF3667; Bio-techne) overnight at 4 °C. After blocking with 1% bovine serum albumin, samples were incubated with anti-DNA antibody for 2 h at room temperature. After washing 3 times, 100 μl of peroxidase substrate was introduced and incubated in darkness for 40 min. The OD value was measured at 405 nm using a microplate reader (ST-960; KHB) to determine NET levels.

### Western blotting

After different treatments, cells were collected and lysed using radioimmunoprecipitation assay lysis buffer (KGB5302-50; Keygen Biotech), followed by centrifugation at 12,000*g* for 10 min at 4 °C. The resulting proteins were separated on 10% sodium dodecyl sulfate–polyacrylamide gel electrophoresis and transferred onto polyvinylidene fluoride membranes. After blocking with 5% bovine serum albumin for 2 h, membranes were incubated overnight at 4 °C with primary antibodies, including anti-IL-6 (1:2,000; 66146-1-Ig; Proteintech), anti-p21 (1:1,000; ab109520; Abcam), anti-CitH3 (1:1,000; ab5103; Abcam), anti-HMGB1 (1:1,000; ab79823; Abcam), anti-β-actin (1:5,000; 66009-1-Ig; Proteintech), and anti-glyceraldehyde-3-phosphate dehydrogenase (GAPDH; 1:5,000; 60004-1-Ig; Proteintech). The membranes were washed and subsequently incubated with secondary antibodies conjugated with horseradish peroxidase for 1 h at 37 °C. Protein bands were visualized using an enhanced chemiluminescence reagent (NCM Biotech). Band intensities were quantified using ImageJ software (National Institutes of Health, USA), and protein expression levels were normalized to β-actin or GAPDH. Each experiment was independently repeated 3 times.

### Flow cytometry

Peripheral blood cells from healthy controls and HCC patients were stained with surface antibodies at room temperature for 30 min. Neutrophils were treated with LPS (10 ng/ml), GM-CSF (100 ng/ml), CU-CPT22 (10 μM; HY-108471; MedChemExpress), TAK-242 (10 μM; HY-11109; MedChemExpress), TH1020 (10 μM; HY-116961; MedChemExpress), hydroxychloroquine (HCQ; 10 μM; HY-W031727; MedChemExpress), glycyrrhizic acid (Gly; 10 μM; HY-N0184; MedChemExpress), and HMGB1 (1 μg/ml; HY-P70570; MedChemExpress) for 6 h, followed by surface staining at room temperature for 30 min. CD8^+^ T cells that had been cocultured for 12 h with either LPS- or GM-CSF-treated (aged) or untreated (non-aged) neutrophils, or stimulated with DNase I (100 IU/ml), NETs (500 ng/ml), or benzyloxycarbonyl-Asp-Glu-Val-Asp-fluoromethyl ketone (Z-DEVD-FMK; 10 μM; HY-12466; MedChemExpress) for 24 h, were fixed and permeabilized after surface staining and incubated overnight at 4 °C for intracellular protein staining. For the CD8^+^ T cell proliferation assay, CD8^+^ T cells were labeled with carboxyfluorescein succinimidyl ester (CFSE; 10 μM; C34570; Invitrogen) by incubation at 37 °C for 20 min in the dark. After removal of excess dye, 5 × 10^5^ CFSE-labeled CD8^+^ T cells were seeded into 24-well plates and cultured under the same experimental conditions as unlabeled CD8^+^ T cells, as described above. All samples were washed with PBS and analyzed by FCM. For apoptosis analysis, HepG2 and HuH7 cells were treated with CM-CD8^+^ T, CM-AT, CM-(AT + Neu), or CM-(AT + AN) and stained with Annexin V/Alexa Fluor 488 and propidium iodide according to the manufacturer’s protocol for the apoptosis detection kit (CA1040; Solarbio). Liver and subcutaneous tumor tissues from C57BL/6 mice were mechanically dissociated through a 200-mesh cell strainer, followed by Percoll density gradient centrifugation. The resulting cell suspensions were stained for membrane proteins at 4 °C for 30 min. Each experiment was independently repeated 3 times.

The antibodies used in the experiments are listed below: fluorophore-conjugated antibodies against human CD3 [peridinin–chlorophyll–protein complex (PerCP)-Cyanine5.5; 45-0037-42; Invitrogen], CD4 [allophycocyanin (APC); 551980; BD Biosciences], CD8 [phycoerythrin (PE); 557086; BD Biosciences], CD66b (PE-Cyanine7; 25-0666-42; Invitrogen), CXCR4 (APC; 17-9999-42; Invitrogen), L-selectin (CD62L) [fluorescein isothiocyanate (FITC); 11-0625-42; Invitrogen], perforin (FITC; 308104; Biolegend), Gzm B (APC; 396408; Biolegend), and Ki67 (FITC; 151212; Biolegend); fluorophore-conjugated antibodies against mouse CD3 (FITC; 11-0032-82; Invitrogen), CD4 (PE; 12-0041-82; Invitrogen), CD8 (APC; 17-0081-82; Invitrogen), Ly6G (FITC; 11-9661-82; Invitrogen), CD11b (PE; 12-0112-82; Invitrogen), CXCR4 (APC; 17-9991-82; Invitrogen), and CD62L (eFluor 450; 48-0621-82; Invitrogen).

### Senescence-associated β-galactosidase staining

Staining of neutrophils treated for 6 h with LPS (10 ng/ml), CU-CPT22 (10 μM), TAK-242 (10 μM ), TH1020 (10 μM), HCQ (10 μM), Gly (10 μM), or HMGB1 (1 μg/ml) was performed using a cellular senescence-associated β-galactosidase (SA-β-Gal) staining kit (C0602; Beyotime). Briefly, cells were incubated overnight at 37 °C in the staining working solution and then observed under a microscope. Each assay was carried out with 3 independent replicates.

### Quantitative real-time polymerase chain reaction

Neutrophils were incubated with or without LPS (10 ng/ml) for 6 h, followed by total RNA extraction. Using total RNA as template, first-strand cDNA was synthesized. The polymerase chain reaction (PCR) amplification protocol was set as follows: initial denaturation at 95 °C for 3 min, followed by 40 cycles of denaturation at 95 °C for 15 s and annealing/extension at 60 °C for 1 min. The mRNA expression levels of *TLR1*, *TLR*2, *TLR4*, *TLR*5, *TLR7*, *TLR8*, and *TLR9* were quantified using a CFX96 real-time PCR system (Bio-Rad) with SYBR Green dye (Biomake) and normalized to GAPDH. All primers were synthesized by Genscript, and their sequences are listed in Table S1. Each assay was carried out with 3 independent replicates.

### Colony-forming unit counting of mouse feces

Fecal samples from ABX-treated and untreated mice were collected into sterile centrifuge tubes containing 1 ml of sterile PBS. After thorough homogenization using a vortex mixer (XH-B, KANG JIAN MEDICAL), the suspensions were serially diluted (1:1,000), and 100 μl of each diluted sample was spread onto tryptic soy agar plates. The plates were incubated at 37 °C for 24 h, and colony-forming units (CFUs) were subsequently counted.

### Single-cell RNA sequencing analysis

The transcriptome data from the tumor cores of 10 HCC patients and 8 adjacent normal tissues were obtained from GSE149614, and those from 16 HCC and 3 adjacent normal tissues were from GSE189903 (https://www.ncbi.nlm.nih.gov/geo/). Cells with unique molecular identifier counts below 500 or above 4,500, or with mitochondrial gene expression exceeding 15% of total RNA, were deemed low quality and excluded from further analysis. Following quality control, the remaining cells underwent logarithmic normalization, and the top 2,000 highly variable genes were identified using the Seurat package (v4.4.0). To eliminate batch effects, the canonical correlation analysis method was applied to integrate multiple Seurat objects into a unified dataset. Dimensionality reduction was then performed using principal components analysis, based on the top 30 principal components. The cellular landscape was visualized using a uniform manifold approximation and projection (UMAP). Finally, all cells were classified into 6 distinct cell types by examining the expression patterns of various cell-specific markers.

### Cell–cell interaction analysis

Cell–cell interaction analysis was conducted using the expression profiles of defined ligand–receptor pairs. Aged neutrophils within the TME were found to interact with various immune cell types. Intercellular communication networks were inferred from gene expression data using the CellChatDB reference database (https://github.com/sqjin/CellChat) for ligand–receptor interactions. To minimize noise from low-quality data, a threshold of a minimum of 3 cells per group was applied to filter out unreliable communication events.

### Whole-transcriptome sequencing analysis

Total RNA was extracted from neutrophils treated with or without LPS (10 ng/ml) for 6 h, followed by mRNA isolation. The captured mRNA was then fragmented, PCR-amplified, and purified using magnetic beads to construct sequencing libraries. After passing quality control, the libraries were sequenced using the Illumina NovaSeq 6,000 platform with paired-end 150–base pair sequencing. Bioinformatic analysis was performed on the raw sequencing data to assess sequencing quality and filter for high-quality reads for further analysis. The quality-controlled reads were aligned to the reference genome, and gene expression levels were quantified based on genome annotation information. Differentially expressed genes (DEGs) were then identified. The RNA-seq data generated in this study have been deposited in the Gene Expression Omnibus (GEO) database and are accessible under accession number GSE326268.

### Kyoto Encyclopedia of Genes and Genomes enrichment analysis

Kyoto Encyclopedia of Genes and Genomes (KEGG) enrichment analysis was performed on the DEGs to explore their potential biological functions and the signaling pathways involved. The enrichment analysis was conducted using the “clusterProfiler” package in R, with the criteria set as a gene count greater than 10 and an adjusted *P* value < 0.050 to define statistical significance.

### Protein–protein interaction network analysis

To elucidate the relationship between NETs and CD8^+^ T cell dysfunction, the Search Tool for the Retrieval of Interacting Genes (STRING, https://cn.string-db.org/) was used. A total of 30 NET-related genes and 102 CD8^+^ T cell exhaustion-related genes in HCC were analyzed [24]. A high-confidence interaction score threshold of ≥0.7 was applied to ensure the reliability of the predicted interactions. The resulting network was then visualized using Cytoscape (version 3.10.2; https://cytoscape.org/).

### Statistical analysis

Data analysis was performed using GraphPad statistical software (version 10.1.2, GraphPad Software Inc.). Spearman correlation analysis was performed to assess the relationship between peripheral blood aged neutrophils and the levels of CD8^+^ T cells, CD4^+^ T cells, serum perforin, and Gzm B in HCC patients. For cellular data, comparisons between 2 groups were conducted using Student’s *t* test, while comparisons among 3 or more groups were evaluated by one-way analysis of variance followed by Newman–Keuls’ multiple comparison test. The Mann–Whitney test was used to determine the clinical significance of peripheral blood aged neutrophils, CD8^+^ T cells, and the CD8^+^ T cell/aged neutrophil ratio across HCC patients with different clinical characteristics. Receiver operating characteristic (ROC) curves were generated to assess the clinical value of aged neutrophils, with the area under the curve (AUC), sensitivity, and specificity calculated using standard methods. Differences were considered statistically significant at *P* < 0.050.

## Results

### Altered abundance of aged neutrophils and their association with CD8^+^ T cells in HCC

**Fig. 1.**
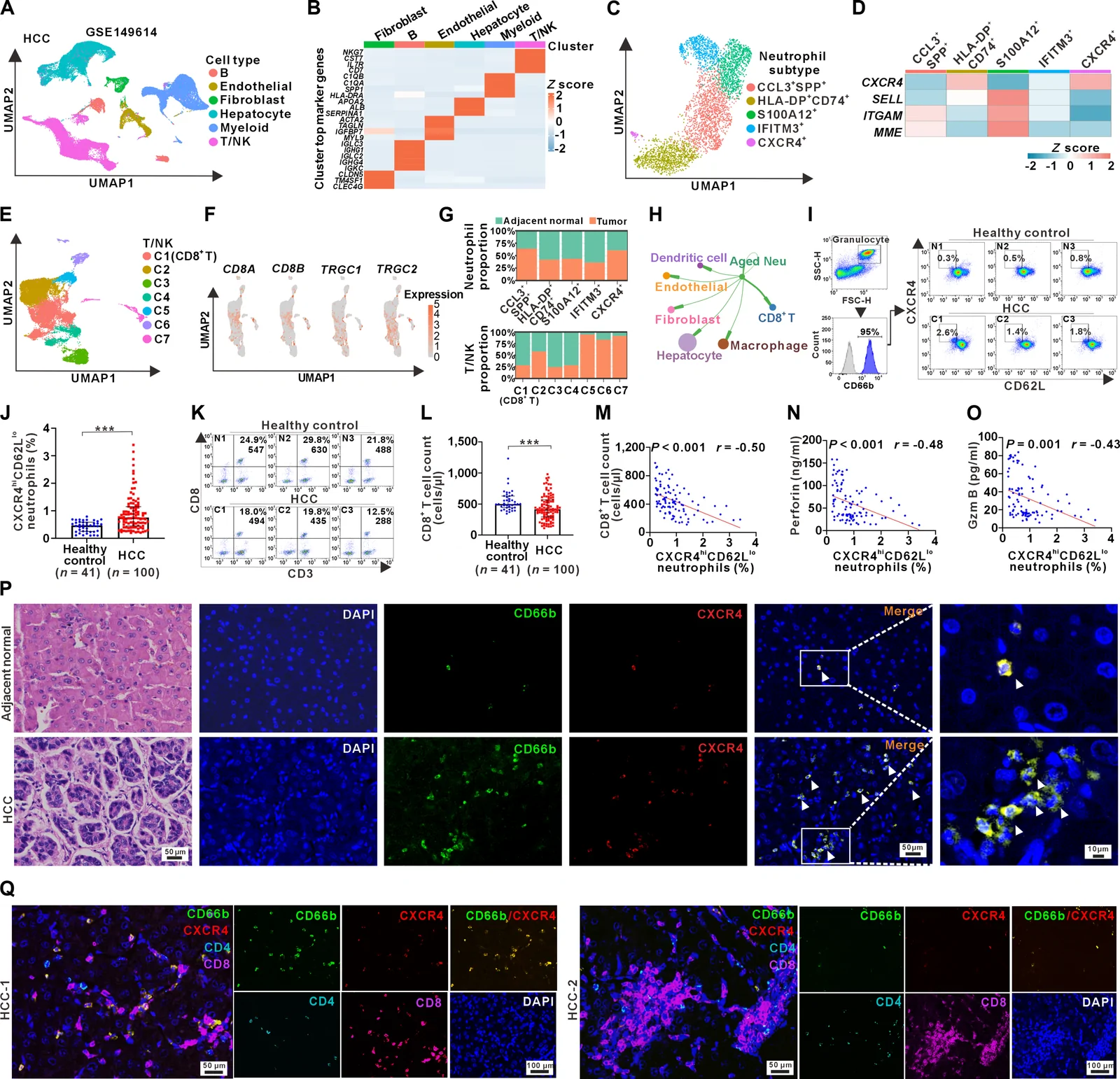
Altered abundance of aged neutrophils and their association with CD8^+^ T cells in HCC. (A) UMAP of 6 cell clusters from the multicellular ecosystem of 10 HCC patients (GSE149614), colored by cell type. (B) Heatmap showing the expression profiles of the top marker genes across different clusters. (C) UMAP of neutrophil subtypes, annotated and colored by clustering. (D) Heatmap showing the aged scores of different neutrophil subtypes. (E) UMAP of T/NK, annotated and colored by clustering. (F) UMAP colored by expression levels (gray to red) of key markers to identify CD8^+^ T clusters. (G) Stacked barplots showing the percentages of neutrophil (top) and T/NK (bottom) clusters in HCC tumor and adjacent normal tissues. (H) Weight of interaction in cell–cell communication network. (I to L) FCM analysis of CXCR4^hi^CD62L^lo^ neutrophil and CD8^+^ T cell count of healthy control (*n* = 41) and HCC (*n* = 100). In (I) and (K), N1 to N3 denote 3 independent healthy control samples, and C1 to C3 denote 3 independent HCC samples. In (K), the numbers shown below the percentage values indicate the absolute counts of CD3^+^CD8^+^ T cells per microliter of peripheral blood. (M to O) Correlation between the percentage of CXCR4^hi^CD62L^lo^ neutrophil and CD8^+^ T cell count, serum levels of perforin, and Gzm B in HCC. (P) IF staining for CD66b and CXCR4 in tumor and adjacent normal tissues from HCC patients. The arrows indicate CD66b^+^CXCR4^+^ neutrophils. (Q) IF staining for CD66b, CXCR4, CD4, and CD8 in tumor tissues from HCC patients. ns, not significant; ***P < 0.001. UMAP, uniform manifold approximation and projection; NK, natural killer cell; Aged Neu, aged neutrophils; HCC, hepatocellular carcinoma; CXCR4^hi^CD62L^lo^, highly expressing CXCR4 and lowly expressing CD62L; Gzm B, granzyme B; DAPI, 4′,6-diamidino-2-phenylindole; FCM, flow cytometry; IF, immunofluorescence.

In this study, the single-cell RNA sequencing (scRNA-seq) dataset GSE149614, which includes transcriptomic profiles from 10 HCC tissues and 8 adjacent normal tissues, was obtained from the GEO database. Following UMAP-based dimensionality reduction and unbiased clustering, the cells were classified into 6 distinct clusters (Fig. 1A). Subsequently, each cluster was annotated and visualized based on key characteristic genes and marker genes (Fig. 1B). Further subclustering of myeloid cells revealed a neutrophil subset (Fig. S1A to C). Subsequent subclustering of neutrophils and T/natural killer (NK) cells identified the CXCR4^+^ neutrophil subset [25] with the change in characteristics of 4 genes [*CXCR4*, selectin L (*SELL*), integrin subunit α M (*ITGAM*), and membrane metalloendopeptidase (*MME*)] as aged neutrophils [26] and enrichment in apoptosis, necroptosis, phagosome, and senescence-related pathways, and the C1 subset with the change in characteristics of 4 genes [*CD8A*, *CD8B*, T cell receptor γ constant 1 (*TRGC1*), and *TRGC2*] as CD8^+^ T cells (Fig. 1C to F and Fig. S1D to F). Representative marker gene expression profiles were quantitatively compared across clusters to validate these biological annotations. HCC tissues displayed a significant increase in the proportion of CXCR4^+^ aged neutrophils alongside a decrease in CD8^+^ T cell proportion, relative to adjacent normal tissues (Fig. 1G). Cell–cell communication analysis using the “CellChat” algorithm indicated that aged neutrophils potentially interact with multiple cell types, including CD8^+^ T cells (Fig. 1H). To validate these findings, we collected peripheral blood specimens from HCC patients and healthy controls for FCM assessment (Table 1). In comparison to healthy controls, HCC patients exhibited an increased frequency of aged neutrophils (CXCR4^hi^CD62L^lo^) in peripheral blood (Fig. 1I and J), accompanied by decreased proportions of CD8^+^ and CD4^+^ T cells (Fig. 1K and L and Fig. S1G). Moreover, aged neutrophil frequency was inversely correlated with CD8^+^ T cell abundance (Fig. 1M) but showed no correlation with CD4^+^ T cells (Fig. S1H). Consistently, higher frequencies of aged neutrophils in peripheral blood were associated with lower concentrations of perforin and Gzm B, 2 key markers of CD8^+^ T cell activation (Fig. 1N and O). IF staining further confirmed increased accumulation of CD66b^+^CXCR4^+^ aged neutrophils in HCC tissues relative to adjacent normal tissues (Fig. 1P). HCC patients with elevated aged neutrophils showed reduced CD8^+^ T cell infiltration compared with patients with lower aged neutrophils (Fig. 1Q). We next performed independent validation of the above findings using an additional publicly available scRNA-seq dataset (GSE189903). Clustering analysis identified a CXCR4^+^ aged neutrophil subset with high *CXCR4* and low *SELL*, *ITGAM*, and *MME* expression (Fig. S2A to G), enriched in phagosome, efferocytosis, apoptosis, and senescence-related pathways (Fig. S2H and I), consistent with neutrophil aging. Following the identification of a CD8^+^ T cell subset through T cell subclustering (Fig. S2J and K), we found a significant negative correlation between CXCR4^+^ aged neutrophils and CD8^+^ T cells in HCC tumor tissues (Fig. S2L). In summary, these results indicated that aged neutrophils were enriched in HCC and inversely correlated with CD8^+^ T cells.

**Table 1.** The clinical characteristics of individuals enrolled for FCM assessment in this study

Characteristic	HCC	Healthy control
Total	100	41
Gender [cases (%)]		
Male	69 (69.0)	25 (61.0)
Female	31 (31.0)	16 (39.0)
Age [cases (%)]		
<60 years	59 (59.0)	36 (87.8)
≥60 years	41 (41.0)	5 (12.2)
Tumor number [cases (%)]		
Solitary	57 (57.0)	Not applicable
Multiple	43 (43.0)	Not applicable
HBV [cases (%)]		
<LLOQ	38 (38.0)	Not available
≥LLOQ	62 (62.0)	Not available
TNM stage [cases (%)]		
I + II	41 (41.0)	Not applicable
III + IV	59 (59.0)	Not applicable
Metastasis [cases (%)]		
Absent	67 (67.0)	Not applicable
Present	33 (33.0)	Not applicable
ALT [U/l; median (interquartile range)]	49.0 (63.3)	32.0 (15.0)
AST [U/l; median (interquartile range)]	42.5 (52.3)	30.0 (16.0)
AFP [μg/l; median (interquartile range)]	298.9 (454.4)	8.7 (21.3)

HCC, hepatocellular carcinoma; *n*, number of samples; HBV, hepatitis B virus; LLOQ, lower limit of quantification; TNM stage (based on the American Joint Committee on Cancer TNM staging system, 8th edition), tumor, node, metastasis staging system; ALT, alanine aminotransferase; AST, aspartate aminotransferase; AFP, α-fetoprotein

### Effects of aged neutrophils on CD8^+^ T cell proliferation and activation

**Fig. 2.**
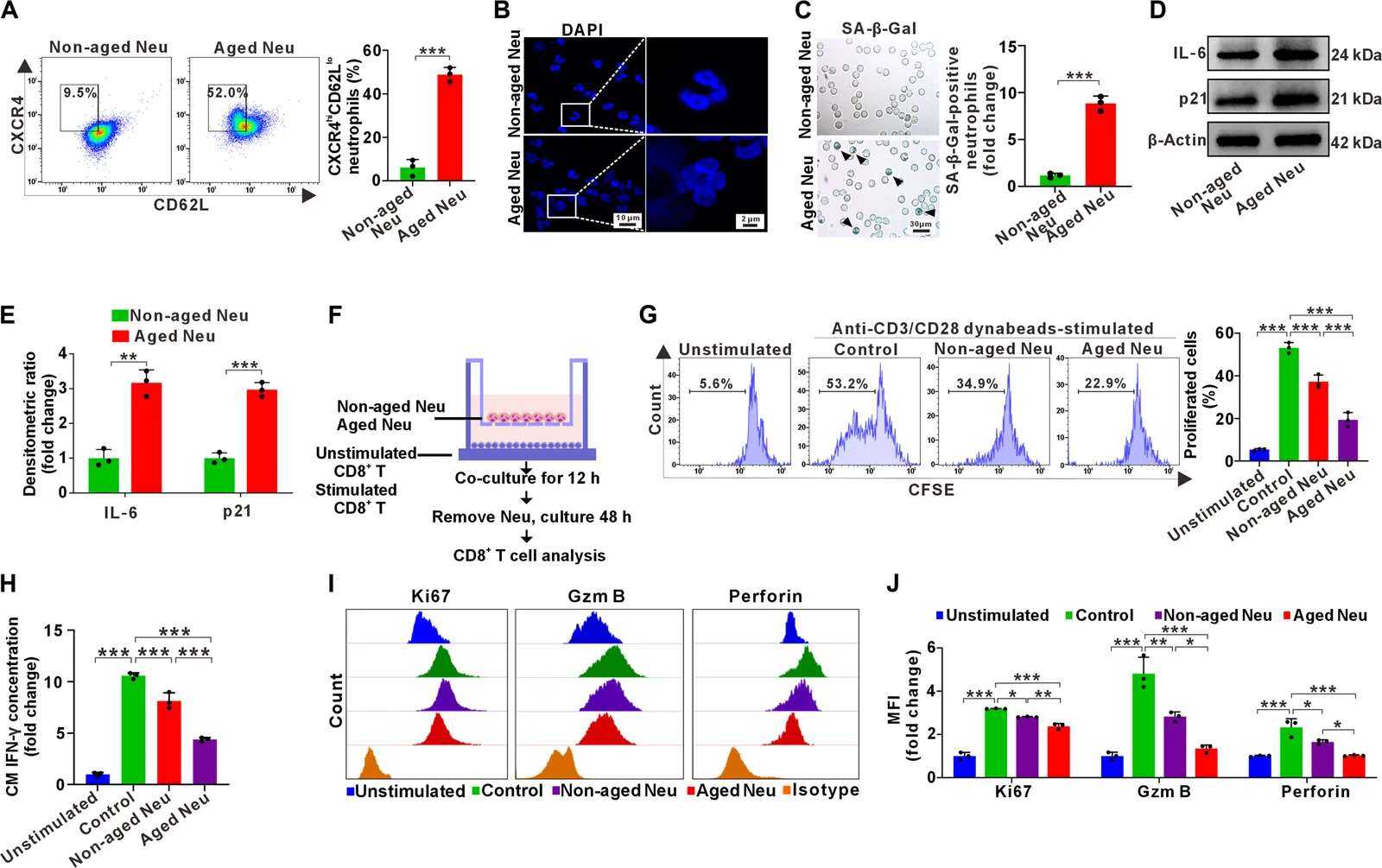
Effects of aged neutrophils on CD8^+^ T cell proliferation and activation. (A) FCM analysis of CXCR4^hi^CD62L^lo^ neutrophils with (Aged Neu group) and without LPS treatment (Non-aged Neu group). (B) Representative fluorescence images of neutrophil nuclei (DAPI staining) with (Aged Neu group) and without LPS treatment (Non-aged Neu group). (C) Representative images and quantification of SA-β-Gal staining with (Aged Neu group) and without LPS treatment (Non-aged Neu group). The arrows indicate SA-β-Gal-positive neutrophils. (D and E) Representative Western blotting and quantification of IL-6 and p21 expression with (Aged Neu group) and without LPS treatment (Non-aged Neu group). (F) Procedure for CD8^+^ T cells analysis by indirect coculture of CD8^+^ T cells with Aged Neu or Non-aged Neu. (G) CFSE analysis of proliferative CD8^+^ T cells cocultured with Aged Neu or Non-aged Neu. (H) ELISA analysis for IFN-γ in CD8^+^ T cell-conditioned medium cocultured with Aged Neu or Non-aged Neu. (I and J) Representative FCM data and MFI quantification of Ki67, Gzm B, and perforin expression in CD8^+^ T cells cocultured with Aged Neu or Non-aged Neu. *P < 0.050, **P < 0.010, ***P < 0.001. FCM, flow cytometry; LPS, lipopolysaccharide; Non-aged Neu, non-aged neutrophils; Aged Neu, aged neutrophils; CXCR4^hi^CD62L^lo^, highly expressing CXCR4 and lowly expressing CD62L; DAPI, 4′,6-diamidino-2-phenylindole; SA-β-Gal, senescence-associated β-galactosidase; kDa, kilodalton; CFSE, carboxyfluorescein succinimidyl ester; ELISA, enzyme-linked immunosorbent assay; CM, conditioned medium; MFI, mean fluorescence intensity; Gzm B, granzyme B.

The following experimental work was undertaken to delineate the influence of aged neutrophils on the functionality of CD8^+^ T cells. Neutrophil senescence was induced by LPS treatment as previously reported [23], characterized by elevated CXCR4 expression, reduced CD62L expression, and hypersegmented nuclei [21]. Isolated human peripheral blood neutrophils were cultured in the presence (Aged Neu group) or absence (Non-aged Neu group) of LPS. FCM analysis demonstrated a higher proportion of CXCR4^hi^CD62L^lo^ neutrophils in the Aged Neu group as opposed to the Non-aged Neu group (Fig. 2A). IF staining further revealed pronounced nuclear hypersegmentation in the Aged Neu group (Fig. 2B). Moreover, SA-β-Gal activity and the expression of senescence-associated secretory phenotype markers IL-6 and p21 were markedly elevated in the Aged Neu group (Fig. 2C to E), confirming successful induction of neutrophil senescence by LPS in vitro. To assess the functional impact on CD8^+^ T cells, aged neutrophils were cocultured with CD8^+^ T cells using a Transwell system (Fig. 2F). Cell proliferation assays based on CFSE showed reduced proliferation of CD8^+^ T cells when cocultured with aged neutrophils (Fig. 2G). This inhibitory effect was further supported by diminished IFN-γ secretion, as quantified by ELISA (Fig. 2H). Additionally, FCM analysis demonstrated significantly lower mean fluorescence intensity (MFI) of Ki67, Gzm B, and perforin in CD8^+^ T cells cocultured with aged neutrophils (Fig. 2I and J). To further validate the above finding that LPS-induced neutrophil aging suppressed CD8^+^ T cells, we applied another aging-inducing factor, GM-CSF, in an in vitro neutrophil culture system. GM-CSF-treated neutrophils showed a significantly increased proportion of aged neutrophils compared with untreated neutrophils (Fig. S3A). When indirectly cocultured with CD8^+^ T cells, aged neutrophils markedly reduced CD8^+^ T cell proliferation, decreased IFN-γ secretion, and the expression of Gzm B and perforin (Fig. S3B to E). Collectively, these observations indicated that aged neutrophils suppressed CD8^+^ T cell proliferation and activation.

### NETs released by aged neutrophils suppressed CD8^+^ T cell proliferation and activation via caspase-3 activation

**Fig. 3.**
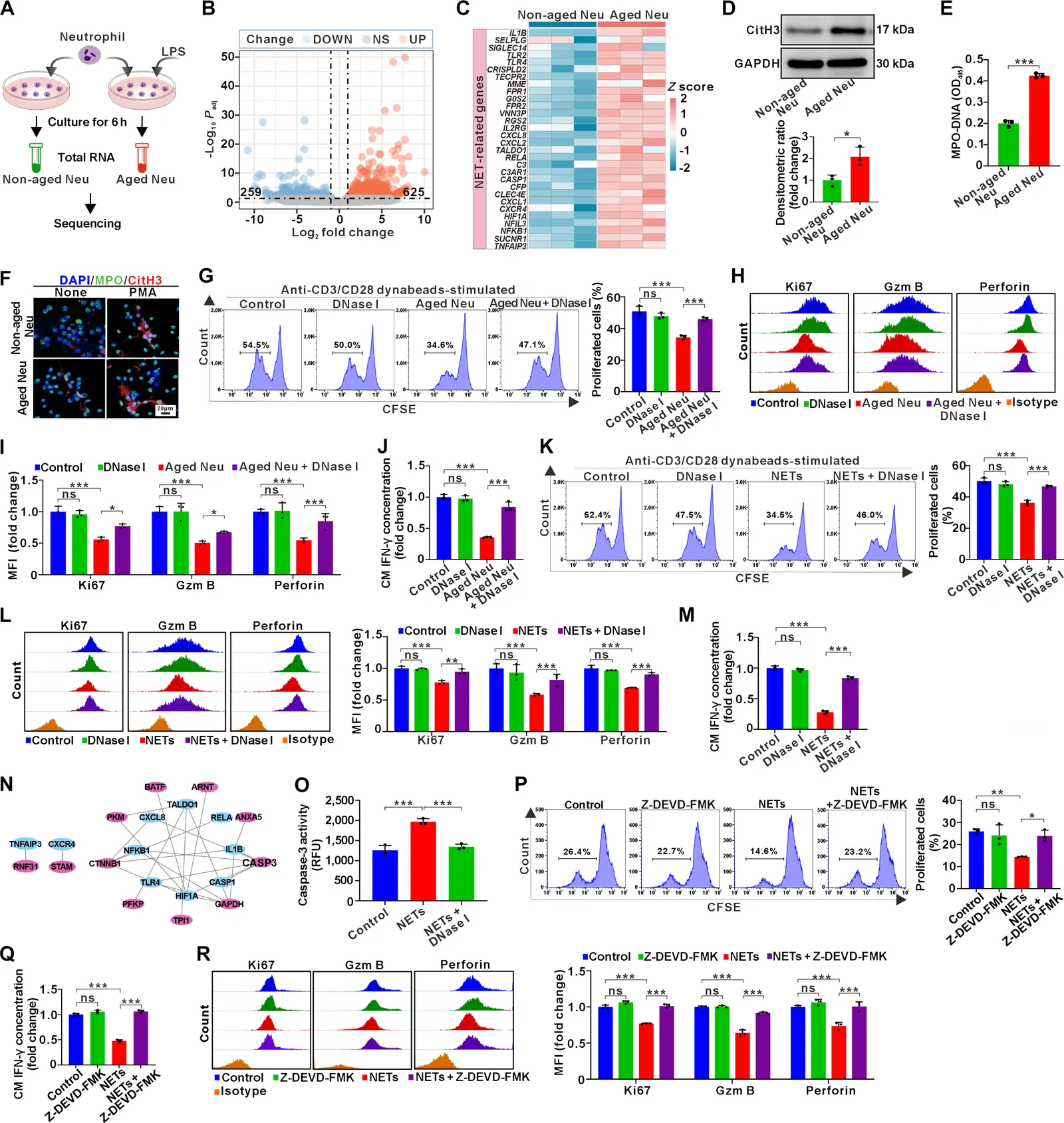
NETs released by aged neutrophils suppressed CD8^+^ T cell proliferation and activation via caspase-3 activation. (A) Procedure for whole-transcriptome sequencing of neutrophils with (Aged Neu group) and without LPS treatment (Non-aged Neu group). (B) Volcano plot of DEGs between Aged Neu and Non-aged Neu. (C) Heatmap of NET-related genes between Aged Neu and Non-aged Neu. (D) Representative Western blotting and quantification of CitH3 expression between Aged Neu and Non-aged Neu. (E) ELISA analysis for MPO–DNA complexes in the culture supernatant of Aged Neu and Non-aged Neu. (F) Representative IF staining of MPO and CitH3 produced by unstimulated or PMA-stimulated Aged Neu and Non-aged Neu. (G) CFSE analysis of CD8^+^ T cell proliferation after DNase I-mediated elimination of NETs released by Aged Neu. (H and I) Representative FCM data and MFI quantification of Ki67, Gzm B, and perforin expression in CD8^+^ T cells after DNase I-mediated elimination of NETs released by Aged Neu. (J) ELISA analysis for IFN-γ in CD8^+^ T cell-conditioned medium after DNase I-mediated elimination of NETs released by Aged Neu. (K) CFSE analysis of CD8^+^ T cell proliferation following treatment with DNase I and/or purified NETs. (L) Representative FCM data and MFI quantification of Ki67, Gzm B, and perforin expression in CD8^+^ T cells following treatment with DNase I and/or purified NETs. (M) ELISA analysis for IFN-γ in CD8^+^ T cell-conditioned medium following treatment with DNase I and/or purified NETs. (N) PPI network showing the interactions of the NET-related genes expressed in aged neutrophils (red) and CD8^+^ T cells exhaustion-related genes (blue). (O) Caspase-3 activity analysis of CD8^+^ T cell following treatment with DNase I and/or purified NETs. (P) CFSE analysis of CD8^+^ T cell proliferation following treatment with Z-DEVD-FMK and/or purified NETs. (Q) ELISA analysis for IFN-γ in CD8^+^ T cell-conditioned medium following treatment with Z-DEVD-FMK and/or purified NETs. (R) Representative FCM data and MFI quantification of Ki67, Gzm B, and perforin expression in CD8^+^ T cells following treatment with Z-DEVD-FMK and/or purified NETs. ns, not significant; *P < 0.050, **P < 0.010, ***P < 0.001. LPS, lipopolysaccharide; Non-aged Neu, non-aged neutrophils; Aged Neu, aged neutrophils; DEGs, differentially expressed genes; *P*_*adj*_, adjusted *P* value; IF, immunofluorescence; MPO, myeloperoxidase; CitH3, citrullinated modification of histone 3; PMA, phorbol 12-myristate 13-acetate; DNase I, deoxyribonuclease I; FCM, flow cytometry; MFI, mean fluorescence intensity; Gzm B, granzyme B; ELISA, enzyme-linked immunosorbent assay; CM, conditioned medium; NETs, neutrophil extracellular traps; PPI, protein–protein interaction; RFU, relative fluorescence unit; Z-DEVD-FMK; benzyloxycarbonyl-Asp-Glu-Val-Asp-fluoromethyl ketone.

We next investigated the molecular mechanisms by which aged neutrophils suppress CD8^+^ T cell proliferation and activation. Neutrophils were cultured with (Aged Neu group) or without LPS stimulation (Non-aged Neu group) for 6 h, followed by total RNA extraction and whole-transcriptome sequencing (Fig. 3A). Among the 884 DEGs, 625 were up-regulated and 259 were down-regulated (Fig. 3B and Table S2). Considering the pivotal role of NETs in aged neutrophils, we focused specifically on NET-related genes (Table S3). Differential gene expression analysis demonstrated that genes linked to NETs were significantly up-regulated in the Aged Neu group versus the Non-aged Neu group (Fig. 3C). To validate the formation of NETs, we examined CitH3 levels by Western blotting, measured MPO–DNA complexes in the culture supernatant using ELISA, and performed IF staining for DNA, MPO, and CitH3. Aged neutrophils expressed higher levels of CitH3 (Fig. 3D) and released higher amounts of MPO–DNA complexes (Fig. 3E). IF staining indicated that relative to the Non-aged Neu group, both unstimulated and PMA-stimulated aged neutrophils released a markedly greater amount of NETs, as evidenced by the increased formation of web-like structures containing MPO and CitH3 (Fig. 3F). The above findings indicated that the formation of NETs was enhanced in aged neutrophils.

The above evidence supported the hypothesis that aged neutrophils might suppress CD8^+^ T cell proliferation and activation through NET release. To test this, we added DNase I to the coculture system of aged neutrophils and CD8^+^ T cells to degrade NETs, and then evaluated CD8^+^ T cell proliferation, indicated by cell proliferation analysis based on CFSE and Ki67 expression, and activation status, reflected by Gzm B, perforin expression, and IFN-γ secretion. NET degradation reversed the suppressive impact of aged neutrophils on CD8^+^ T cell proliferation and activation (Fig. 3G to J). Moreover, direct exposure of CD8^+^ T cells to purified NETs markedly reduced the frequency of proliferating cells and attenuated the levels of Ki67, Gzm B, perforin, and IFN-γ (Fig. 3K to M), indicating that NETs alone may impair CD8^+^ T cell proliferative capacity and effector functions. This suppressive effect was also reversed by cotreatment with DNase I (Fig. 3K to M), further confirming the involvement of NETs in driving CD8^+^ T cell inhibition.

We then further explored the underlying mechanism of this immunosuppressive effect. Protein–protein interaction (PPI) network analysis revealed that caspase-3, encoded by the *caspase 3* gene in CD8^+^ T cells, potentially interacted with multiple NET-associated proteins encoded by genes highly expressed in aged neutrophils (Fig. 3N and Tables S3 and S4), indicating that *caspase 3* may integrate signals from a broader network of NET-related pathways. Based on this, we investigated whether NETs suppress CD8^+^ T cell proliferation and activation by modulating caspase-3 activity. Direct exposure of CD8^+^ T cells to purified NETs led to a marked increase in caspase-3 activity relative to both the control and NETs + DNase I groups (Fig. 3O). To determine the function of caspase-3, the system was exposed to Z-DEVD-FMK, a selective caspase-3 inhibitor. Z-DEVD-FMK treatment alone did not markedly alter CD8^+^ T cell proliferation or activation (Fig. 3P to R). However, cotreatment with NETs and Z-DEVD-FMK markedly restored CD8^+^ T cell proliferation and up-regulated Ki67, Gzm B, perforin, and IFN-γ expression (Fig. 3P to R). These results indicated that caspase-3 inhibition effectively reversed the NET-mediated suppression of CD8^+^ T cell function.

Together, these results revealed that aged neutrophils promoted NET formation, which in turn increased caspase-3 activity in CD8^+^ T cells and consequently suppressed their proliferation and activation.

### Impact of aged neutrophils on CD8^+^ T cell-mediated regulation of HCC cell behavior in vitro

**Fig. 4.**
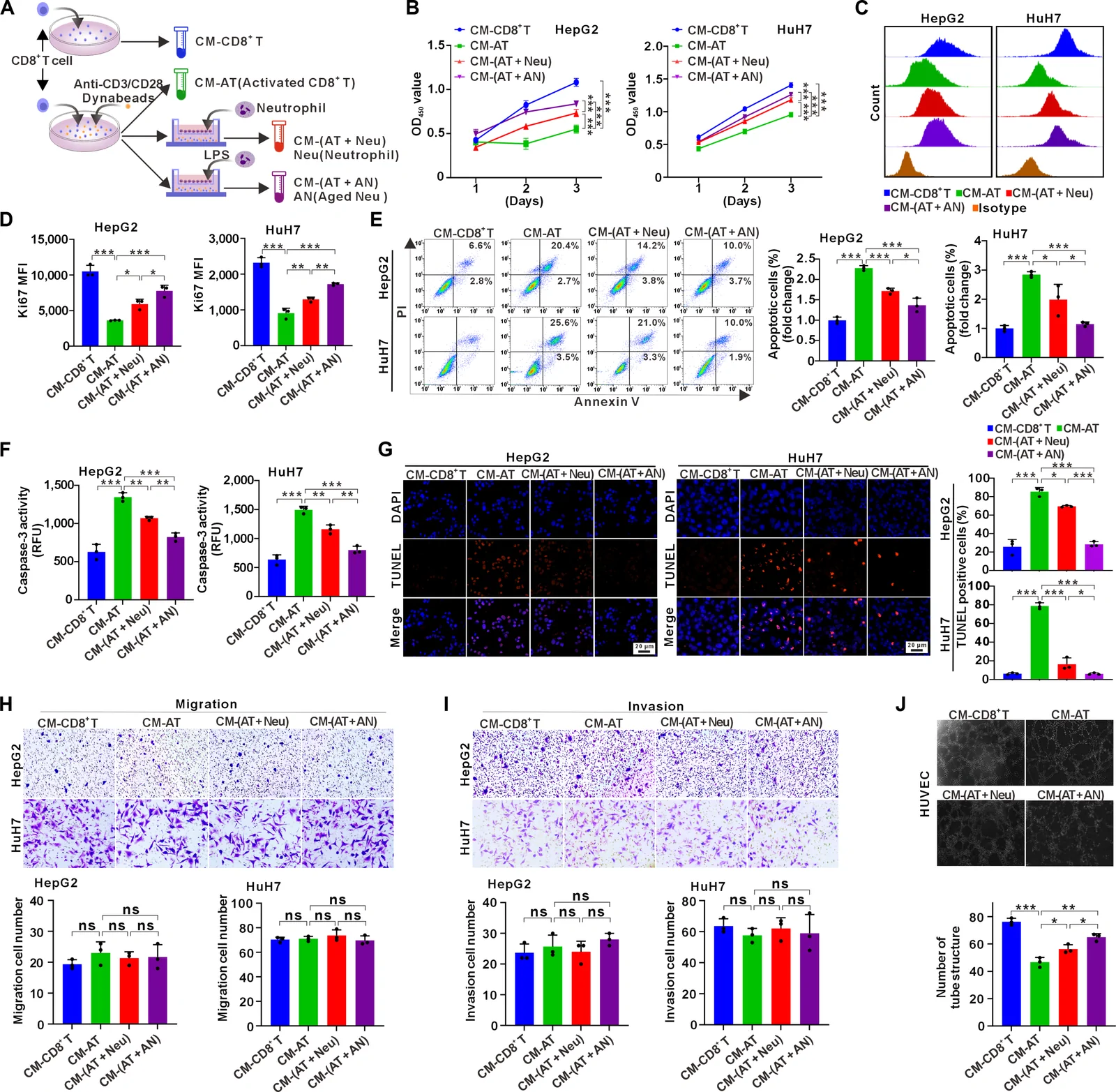
Impact of aged neutrophils on CD8^+^ T cell-mediated regulation of HCC cell behavior in vitro*.* (A) Schematic diagram of CM collection from CD8^+^ T cells cocultured with non-aged neutrophils, referred to as CM-(AT + Neu), or aged neutrophils, referred to as CM-(AT + AN). (B) CCK-8 analysis for cell survival of HepG2 and HuH7 cells treated with CM-CD8^+^ T, CM-AT, CM-(AT + Neu), or CM-(AT + AN) for sequential 3 d. (C and D) Representative FCM data and MFI quantification of Ki67 expression in HepG2 and HuH7 cells treated with CM-CD8^+^ T, CM-AT, CM-(AT + Neu), or CM-(AT + AN). (E) Apoptosis analysis for HepG2 and HuH7 cells treated with CM-CD8^+^ T, CM-AT, CM-(AT + Neu), or CM-(AT + AN). (F) Caspase-3 activity analysis of HepG2 and HuH7 cells treated with CM-CD8^+^ T, CM-AT, CM-(AT + Neu), or CM-(AT + AN). (G) TUNEL staining of HepG2 and HuH7 cells treated with CM-CD8^+^ T, CM-AT, CM-(AT + Neu), or CM-(AT + AN). (H) Transwell migration assay for HepG2 and HuH7 cells treated with CM-CD8^+^ T, CM-AT, CM-(AT + Neu), or CM-(AT + AN). (I) Transwell invasion assay for HepG2 and HuH7 cells treated with CM-CD8^+^ T, CM-AT, CM-(AT + Neu), or CM-(AT + AN). (J) Matrigel tube formation assay for HUVEC treated with CM-CD8^+^ T, CM-AT, CM-(AT + Neu), or CM-(AT + AN). ns, not significant; *P < 0.050, **P < 0.010, ***P < 0.001. LPS, lipopolysaccharide; CM, conditioned medium; AT, avtivated CD8^+^ T; Neu, neutrophils; Aged Neu, aged neutrophils; CCK-8, cell counting kit-8; FCM, flow cytometry; MFI, mean fluorescence intensity; PI, propidium iodide; RFU, relative fluorescence unit; TUNEL, terminal deoxynucleotidyl transferase deoxyuridine triphosphate nick end labeling; DAPI, 4′,6-diamidino-2-phenylindole.

We collected CM from CD8^+^ T cells maintained together with non-aged neutrophils or aged neutrophils, referred to as CM-(AT + Neu) and CM-(AT + AN), respectively (Fig. 4A), and applied them to HepG2 and HuH7 cells to evaluate changes in malignant behaviors, including proliferation, apoptosis, migration, invasion, and angiogenesis. CCK-8 and FCM analyses demonstrated that, relative to CM-(AT + Neu), CM-(AT + AN) significantly increased cell viability and elevated Ki67 expression in both HepG2 and HuH7 cell lines (Fig. 4B to D), suggesting that aged neutrophils attenuated the suppressive effect of CD8^+^ T cells on the proliferation of HCC cells in vitro. Apoptotic cells were quantified by Annexin V and propidium iodide staining, terminal deoxynucleotidyl transferase deoxyuridine triphosphate nick end labeling (TUNEL) assay (to detect DNA fragmentation), and measurement of caspase-3 activity using a fluorescence microplate reader. CM-(AT + AN) treatment markedly decreased apoptotic cell proportions (Fig. 4E), suppressed caspase-3 activity (Fig. 4F), and reduced TUNEL-positive cells (Fig. 4G) in both HepG2 and HuH7 cell lines compared with CM-(AT + Neu), indicating that aged neutrophils attenuated the pro-apoptotic effect of CD8^+^ T cells on HCC cells in vitro. Subsequently, transwell chamber assays demonstrated no significant alterations in migration or invasion of HepG2 and HuH7 cells after exposure to CM from CD8^+^ T cells cultured in the presence of aged neutrophils (Fig. 4H and I). Angiogenic potential was assessed by tube formation assays using HUVECs, showing that CM-(AT + AN) significantly enhanced tubular structure formation relative to CM-(AT + Neu) (Fig. 4J), suggesting promotion of angiogenesis by CM from CD8^+^ T cells cocultured with aged neutrophils. Collectively, these results indicated that aged neutrophils altered CD8^+^ T cell-mediated regulation of HCC cell behaviors in vitro.

### Aged neutrophils accelerated HCC progression via CD8^+^ T cell dysfunction in vivo

**Fig. 5.**
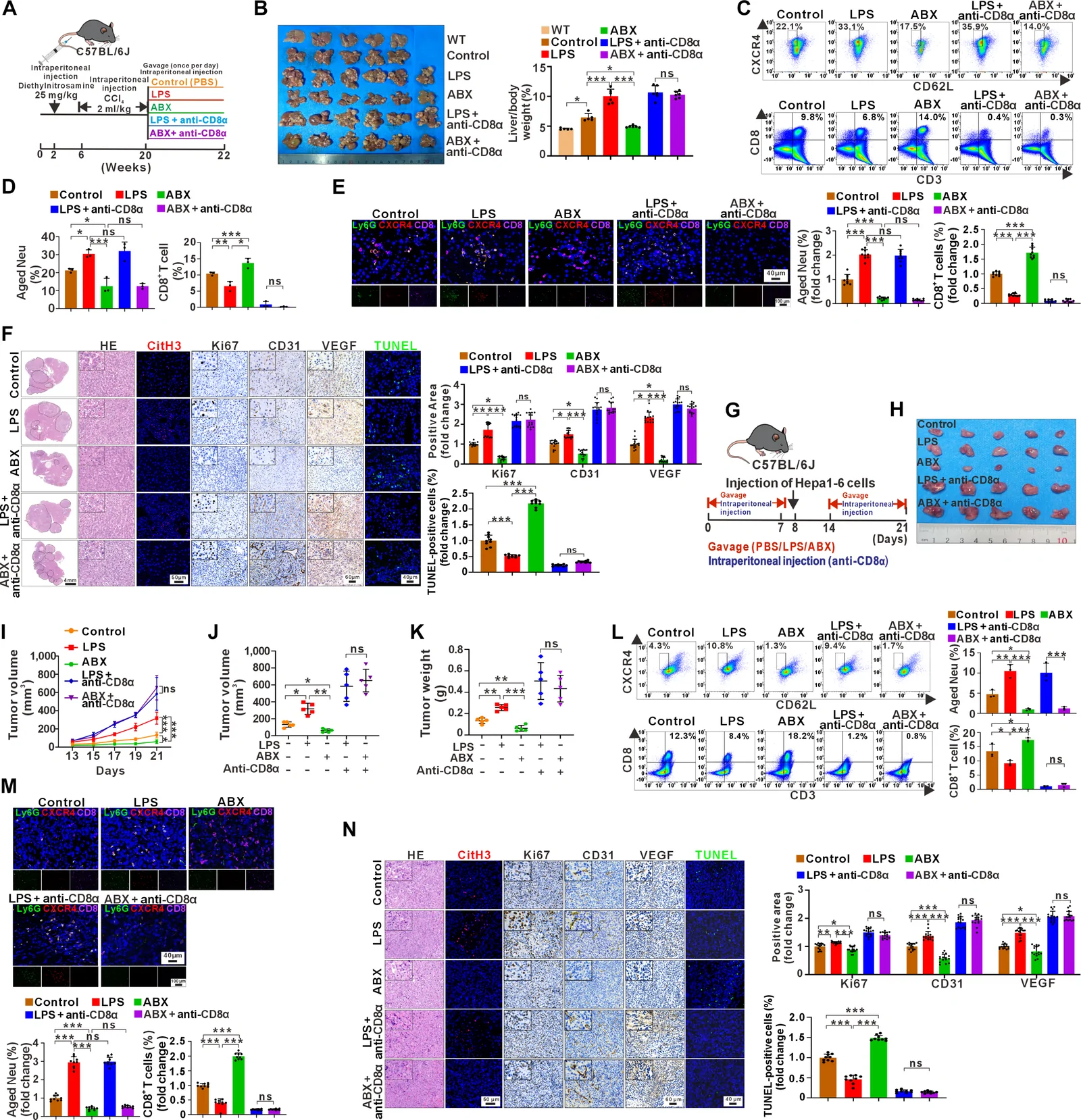
Aged neutrophils accelerated HCC progression via CD8^+^ T cell dysfunction in vivo. (A) Schematic diagram of the orthotopic HCC mouse model induced by diethylnitrosamine and CCl_4_. (B) Representative liver images and liver/body weight ratio analysis from untreated WT mice and diethylnitrosamine and CCl_4_-induced orthotopic HCC mice receiving different treatments: Control, LPS, ABX, LPS + anti-CD8α, and ABX + anti-CD8α. (C and D) FCM analysis of tumor-infiltrating CXCR4^hi^CD62L^lo^ neutrophils and CD8^+^ T cells in diethylnitrosamine and CCl_4_-induced orthotopic HCC mice receiving different treatments: Control, LPS, ABX, LPS + anti-CD8α, and ABX + anti-CD8α. (E) IF staining for Ly6G, CXCR4, and CD8 in tumor sections and quantification of the percentages of Ly6G^+^CXCR4^+^ and CD8^+^ T cells from diethylnitrosamine and CCl_4_-induced orthotopic HCC mice receiving different treatments: Control, LPS, ABX, LPS + anti-CD8α, and ABX + anti-CD8α. (F) Representative images and quantification of hematoxylin and eosin (H&E) staining, IHC staining for Ki67, CD31, and VEGF, TUNEL staining, and IF staining for CitH3 in tumor sections from diethylnitrosamine and CCl_4_-induced orthotopic HCC mice receiving different treatments: Control, LPS, ABX, LPS + anti-CD8α, and ABX + anti-CD8α. (G) Schematic diagram of the syngeneic HCC transplantation model established by subcutaneous injection of Hepa 1-6 cells. Mice were subjected to different treatments: Control, LPS, ABX, LPS + anti-CD8α, and ABX + anti-CD8α. (H) Images of bearing tumors from syngeneic HCC transplantation mice receiving different treatments: Control, LPS, ABX, LPS + anti-CD8α, and ABX + anti-CD8α. (I) Tumor growth curve in syngeneic HCC transplantation mice receiving different treatments: Control, LPS, ABX, LPS + anti-CD8α, and ABX + anti-CD8α. Subcutaneous tumor growth was assessed at 2-d intervals. (J and K) Tumor volume and weight analysis in syngeneic HCC transplantation mice receiving different treatments: Control, LPS, ABX, LPS + anti-CD8α, and ABX + anti-CD8α. (L) FCM analysis of tumor-infiltrating CXCR4^hi^CD62L^lo^ neutrophils and CD8^+^ T cells in the syngeneic HCC transplantation model. (M) IF staining for Ly6G, CXCR4, and CD8 in syngeneic tumor sections and quantification of the percentages of Ly6G^+^CXCR4^+^ and CD8^+^ T cells. (N) Representative images and quantification of H&E staining, IHC staining for Ki67, CD31, and VEGF, TUNEL staining, and IF staining for CitH3 in syngeneic tumor sections. ns, not significant; *P < 0.050, **P < 0.010, ***P < 0.001. CCl_4_, carbon tetrachloride; PBS, phosphate-buffered saline; WT, wild type; LPS, lipopolysaccharide; ABX, broad-spectrum antibiotic cocktail; Aged Neu, aged neutrophils; FCM, flow cytometry; IF, immunofluorescence; H&E, hematoxylin and eosin; IHC, immunohistochemistry; VEGF, vascular endothelial growth factor; TUNEL, terminal deoxynucleotidyl transferase deoxyuridine triphosphate nick end labeling; CitH3, citrullinated modification of histone 3.

To examine the contribution of aged neutrophils to HCC development in vivo, we established a long-term orthotopic HCC model in wild-type (WT) mice via intraperitoneal injection of diethylnitrosamine and CCl_4_. Subsequently, we administered LPS by oral gavage to enrich aged neutrophils in vivo, and used a broad-spectrum ABX to reduce gut microbiota abundance, thereby reducing the levels of aged neutrophils (Fig. S4A). In addition, intraperitoneal injection of an anti-CD8α antibody was performed to deplete CD8^+^ T cells in vivo (Fig. 5A). LPS administration resulted in elevated tumor burden and liver-to-body weight ratios, while ABX treatment produced the opposite effect relative to control mice (Fig. 5B). In contrast, no significant difference was observed in the liver-to-body weight ratio between the LPS + anti-CD8α and ABX + anti-CD8α groups, in which CD8^+^ T cells were depleted, suggesting that this effect was CD8^+^ T cell dependent (Fig. 5B). We further analyzed the proportion of tumor-infiltrating CXCR4^hi^CD62L^lo^ aged neutrophils and CD8^+^ T cells in the orthotopic HCC tissues. The frequency of CXCR4^hi^CD62L^lo^ aged neutrophils was markedly increased in the LPS group and markedly decreased in the ABX group (Fig. 5C and D). Meanwhile, CD8^+^ T cell infiltration was dramatically reduced in mice treated with the anti-CD8α antibody (Fig. 5C and D), confirming the effectiveness of the model intervention. Notably, relative to controls, CD8^+^ T cell infiltration and serum Gzm B and perforin levels were reduced in LPS-treated mice but elevated in the ABX group (Fig. 5C and D and Fig. S4B and C). IF staining further confirmed that the LPS group exhibited increased infiltration of Ly6G^+^CXCR4^+^ aged neutrophils and reduced infiltration of CD8^+^ T cells in HCC tissues, whereas the ABX group showed the opposite trend (Fig. 5E). CitH3 and MPO–DNA levels followed a pattern similar to aged neutrophil infiltration (Fig. 5F and Fig. S4D). Collectively, these results indicated that aged neutrophils could facilitate HCC progression by modulating CD8^+^ T cell responses. We next performed IHC staining of the proliferation marker Ki67 and angiogenesis-related factors CD31 and VEGF, as well as TUNEL staining to assess apoptosis in HCC tissues. The LPS group showed significantly increased expression of Ki67, CD31, and VEGF, along with a marked reduction in TUNEL-positive cells, whereas the ABX group displayed the opposite trend. Notably, no significant differences were observed between the LPS + anti-CD8α and ABX + anti-CD8α groups (Fig. 5F), further supporting the notion that aged neutrophils promoted tumor cell proliferation and angiogenesis and inhibited apoptosis, likely through modulation of CD8^+^ T cells.

To validate the generalizability of the above findings, we further established a subcutaneous HCC allograft model (Fig. 5G). Fecal CFU counting confirmed the effectiveness of ABX treatment in depleting gut microbiota (Fig. S4E). Consistent with previous results, LPS administration promoted tumor growth, whereas ABX treatment significantly suppressed tumor burden, volume, and weight (Fig. 5H to K). Upon CD8^+^ T cell depletion, no significant differences were observed between the LPS + anti-CD8α and ABX + anti-CD8α groups (Fig. 5I to K), further supporting that this effect was CD8^+^ T cell-dependent. FCM and IF analyses also revealed that LPS increased the intratumoral infiltration of aged neutrophils while reducing CD8^+^ T cell infiltration as well as serum Gzm B and perforin levels, whereas ABX exerted the opposite effect (Fig. 5L and M and Fig. S4F and G). The NET markers CitH3 and MPO–DNA exhibited expression similar to those of infiltrating aged neutrophils (Fig. 5N and Fig. S4H). In addition, tumors from the LPS-treated group exhibited increased expression of Ki67, CD31, and VEGF, along with a reduced proportion of TUNEL-positive cells, while ABX treatment led to opposite changes (Fig. 5N). These differences were abolished upon CD8^+^ T cell depletion (Fig. 5N).

Taken together, aged neutrophils accelerated HCC progression in vivo by inducing CD8^+^ T cell dysfunction.

### In vivo assessment of HCC cell metastasis under aged neutrophil-associated CD8^+^ T cell dysfunction

**Fig. 6.**
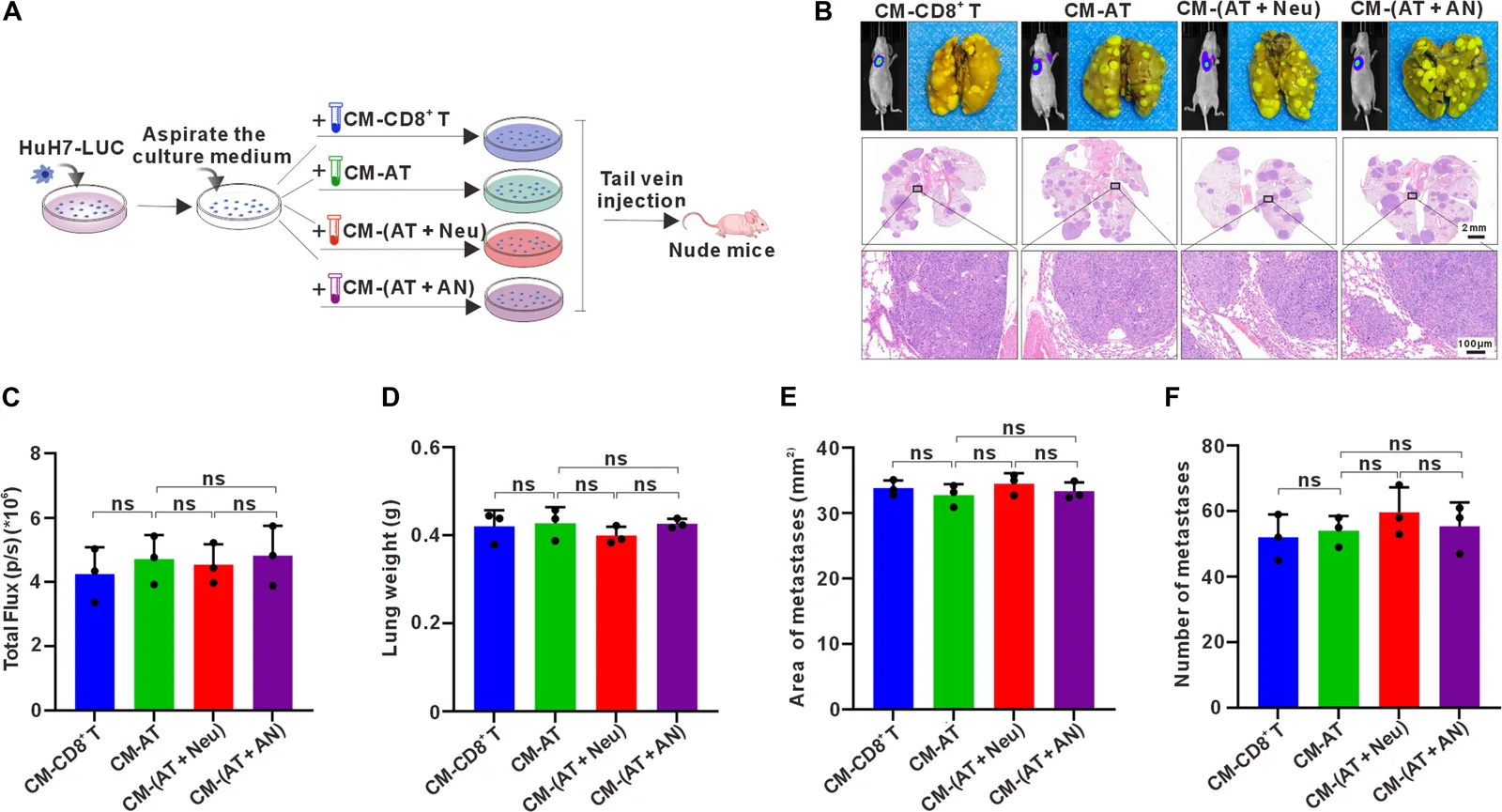
In vivo assessment of HCC cell metastasis under aged neutrophil-associated CD8^+^ T cell dysfunction. (A) Schematic diagram of the HCC metastasis mouse model induced by HuH7-LUC cells with various treatments. (B) Representative images of bioluminescence imaging, gross lung morphology, and H&E-stained lung sections from the HuH7-LUC-induced lung metastasis model. (C) Total flux analysis of bioluminescence imaging from the HCC metastasis mouse model induced by HuH7-LUC cells with various treatments. (D) Lung weight of the HCC metastasis mouse model induced by HuH7-LUC cells with various treatments. (E) Metastatic area in the HCC metastasis mouse model induced by HuH7-LUC cells with various treatments. (F) Number of metastatic foci in the HCC metastasis mouse model induced by HuH7-LUC cells with various treatments. ns, not significant. HuH7-LUC; HuH7 cell line stably expressing luciferase; CM, conditioned medium; AT, activated CD8^+^ T cells; Neu, neutrophils; AN, aged neutrophils; H&E, hematoxylin and eosin.

To further investigate tumor metastasis in vivo, we established a metastatic model by injecting HuH7-LUC cells into mice via the tail vein. Prior to injection, HuH7-LUC cells were pretreated with CM collected from CD8^+^ T cells cocultured with non-aged neutrophils or aged neutrophils, referred to as CM-(AT + Neu) and CM-(AT + AN), respectively (Fig. 6A). Metastatic progression and tumor burden were monitored by bioluminescence imaging. At day 30 post-injection, mice were euthanized for organ collection and histopathological analysis to determine the presence of metastatic lesions in the lungs (Fig. 6B). Luciferase activity derived from HuH7-LUC cells in vivo was assessed by intraperitoneal injection of d-luciferin. The total photon flux in the thoracic region showed no significant difference among the treatment groups (Fig. 6C), suggesting similar tumor cell colonization efficiency. To further evaluate tumor burden, we analyzed lung tissue weight, metastatic area, and the number of metastatic nodules. Compared with the CM-(AT + Neu) group, HuH7-LUC cells treated with CM-(AT + AN) showed no significant difference in lung tumor burden in this metastasis model (Fig. 6D to F). These findings were consistent with our in vitro results, indicating that the dysfunction of CD8^+^ T cells modulated by aged neutrophils did not affect the metastatic potential of HCC cells in vivo.

### HMGB1 derived from HCC cells promoted neutrophil senescence via the TLR4 signaling axis

**Fig. 7.**
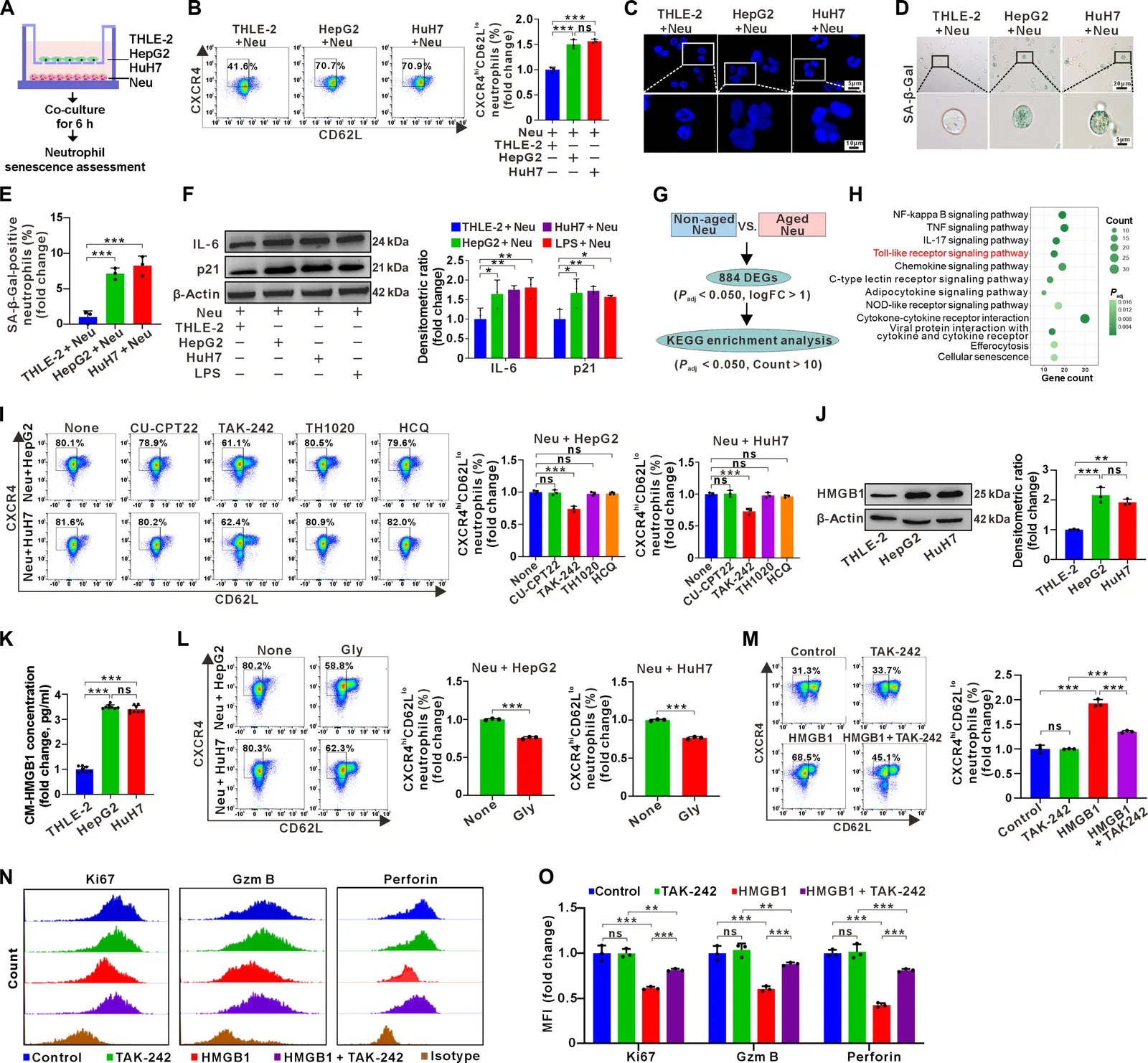
HMGB1 derived from HCC cells promoted neutrophil senescence via the TLR4 signaling axis. (A) Procedure for neutrophil senescence analysis by indirect coculture of neutrophil with THLE-2, HepG2, or HuH7 for 6 h. (B) FCM analysis of CXCR4^hi^CD62L^lo^ neutrophils following indirect coculture of neutrophil with THLE-2, HepG2, or HuH7. (C) Representative fluorescence images of neutrophil nuclei (DAPI staining) following indirect coculture of neutrophil with THLE-2, HepG2, or HuH7. (D and E) Representative images and quantification of SA-β-Gal staining following indirect coculture of neutrophil with THLE-2, HepG2, or HuH7. (F) Representative Western blotting and quantification of IL-6 and p21 expression following indirect coculture of neutrophil with THLE-2, HepG2, or HuH7, or after LPS-induced neutrophil senescence. (G) Schematic diagram of KEGG enrichment analysis based on DEGs between Aged Neu and Non-aged Neu. (H) KEGG pathway enrichment analysis based on DEGs between Aged Neu and Non-aged Neu. The bubble size corresponds to the number of involved genes, and the color gradient represents the significance level. (I) FCM analysis of CXCR4^hi^CD62L^lo^ neutrophils following coculture of neutrophil with HepG2 or HuH7 cells and treatment with CU-CPT22, TAK-242, TH1020, or HCQ. (J) Representative Western blotting and quantification of HMGB1 expression in THLE-2, HepG2, and HuH7 cells. (K) ELISA analysis for HMGB1 in CM from THLE-2, HepG2, and HuH7 cells. (L) FCM analysis of CXCR4^hi^CD62L^lo^ neutrophils following coculture of neutrophil with HepG2 or HuH7 cells and treatment with Gly. (M) FCM analysis of CXCR4^hi^CD62L^lo^ neutrophils following treatment with HMGB1 and/or TAK-242. (N and O) Representative FCM data and MFI quantification of Ki67, Gzm B, and perforin expression in CD8^+^ T cells cocultured with neutrophils treated with HMGB1 and/or TAK-242. ns, not significant; *P < 0.050, **P < 0.010, ***P < 0.001. HMGB1, high mobility group box 1; TLR4, Toll-like receptor 4; Neu, neutrophils; CXCR4^hi^CD62L^lo^, highly expressing CXCR4 and lowly expressing CD62L; DAPI, 4′,6-diamidino-2-phenylindole; SA-β-Gal, senescence-associated β-galactosidase; LPS, lipopolysaccharide; kDa, kilodalton; Non-aged Neu, non-aged neutrophils; Aged Neu, aged neutrophils; DEGs, differentially expressed genes; *P*_*adj*_, adjusted *P* value; KEGG, Kyoto Encyclopedia of Genes and Genomes; FCM, flow cytometry; HCQ, hydroxychloroquine; CM, conditioned medium; ELISA, enzyme-linked immunosorbent assay; Gly, glycyrrhizic acid; MFI, mean fluorescence intensity; Gzm B, granzyme B.

To investigate whether HCC cells induce neutrophil senescence, we performed indirect coculture of neutrophils with THLE-2, HepG2, or HuH7 (Fig. 7A). Neutrophil senescence was assessed by evaluating the expression of CXCR4 and CD62L, nuclear hypersegmentation, SA-β-Gal activity, and the protein levels of IL-6 and p21. Compared with neutrophils cocultured with THLE-2, those exposed to HepG2 or HuH7 cells exhibited a significantly higher proportion of CXCR4^hi^CD62L^lo^ aged neutrophils (Fig. 7B), more pronounced nuclear hypersegmentation (Fig. 7C), an increase in SA-β-Gal-positive cells (Fig. 7D and E), and elevated levels of IL-6 and p21 (Fig. 7F). To explore the underlying molecular mechanisms, we conducted KEGG pathway enrichment analysis on DEGs of aged and non-aged neutrophils (Fig. 7G and Table S5). The results revealed enrichment of multiple signaling pathways, including the TLR pathway (Fig. 7H). Given that a previous study by others have demonstrated a close association between gut microbiota-driven neutrophil senescence and TLR signaling [19], we therefore focused on investigating whether TLR-related signaling is involved in this mechanism. PCR validation confirmed that among the TLR family members, *TLR4* expression was the most significantly up-regulated in aged neutrophils (Fig. S5A). To determine which specific TLR contributes to neutrophil senescence, we added inhibitors targeting different TLRs to the coculture system of neutrophils with HepG2 or HuH7. Only TLR4 inhibitor TAK-242 significantly reduced the proportion of CXCR4^hi^CD62L^lo^ aged neutrophils, whereas inhibitors targeting TLR1/2 (CU-CPT22), TLR5 (TH1020), and TLR7/9 (HCQ) had no such effects (Fig. 7I). Consistent results were observed in nuclear morphology and SA-β-Gal assays (Fig. S5B and C).

Previous studies have shown that HMGB1, a known ligand of TLR4, participates in regulating cellular senescence across multiple cell types, including melanoma cells [27] and bone marrow mesenchymal stem cells [28]. Thus, we selected HMGB1 as the target for further investigation. Western blotting and ELISA results confirmed that HepG2 and HuH7 cells showed a significant increase in HMGB1 expression compared with THLE-2 cells (Fig. 7J and K). Addition of the HMGB1 antagonist Gly to the coculture system significantly reduced the proportion of CXCR4^hi^CD62L^lo^ aged neutrophils (Fig. 7L) and SA-β-Gal-positive cells (Fig. S5D). In contrast, treatment with Gly in a neutrophil-only culture system had no significant effect on CXCR4^hi^CD62L^lo^ aged neutrophil proportion or SA-β-Gal activity compared to untreated controls (Fig. S5E and F), suggesting that Gly did not directly affect neutrophil senescence but acted by antagonizing HMGB1. Further confirmation was obtained by adding exogenous recombinant HMGB1 protein to the neutrophil-only culture system. HMGB1 treatment significantly increased the proportion of CXCR4^hi^CD62L^lo^ aged neutrophils (Fig. 7M) and SA-β-Gal-positive cells (Fig. S5G), indicating that HMGB1 promoted neutrophil senescence. Notably, TAK-242 alone had no significant effect on neutrophils, but cotreatment with HMGB1 and TAK-242 effectively reversed HMGB1-induced neutrophil senescence (Fig. 7M and Fig. S5G), further supporting the conclusion that HMGB1 drove neutrophil senescence by activating TLR4 signaling. In addition, FCM analysis revealed that the MFI of Ki67, Gzm B, and perforin was significantly reduced in CD8^+^ T cells cocultured with HMGB1-induced aged neutrophils (Fig. 7N and O). However, these reductions were restored when CD8^+^ T cells were cocultured with HMGB1-induced aged neutrophils in the presence of TAK-242 (Fig. 7N and O). To assess the role of HMGB1–TLR4 signaling-mediated neutrophil aging in HCC progression in vivo, we established a subcutaneous syngeneic HCC mouse model (Fig. S5H). Intratumoral Hmgb1 markedly increased tumor volume and weight, which were significantly alleviated by cotreatment with Gly or TAK-242 (Fig. S5I to L). IF staining revealed increased intratumoral Ly6G^+^CXCR4^+^ aged neutrophils and concomitantly decreased CD8^+^ T cells after Hmgb1 treatment, whereas Gly or TAK-242 reduced aged neutrophil accumulation and restored CD8^+^ T cell function (Fig. S5M). The NET marker CitH3 showed a similar pattern of expression as aged neutrophil infiltration (Fig. S5N). Hmgb1 also up-regulated Ki67, CD31, and VEGF while reducing TUNEL-positive cells; these pro-proliferative and pro-angiogenic effects were suppressed by Gly or TAK-242, accompanied by increased tumor cell apoptosis (Fig. S5N).

Taken together, HMGB1 derived from HCC cells promoted neutrophil senescence via TLR4 signaling, resulting in impaired CD8^+^ T cell function and enhanced HCC progression in vivo.

### The clinical importance of detecting circulating aged neutrophils in HCC patients

**Fig. 8.**
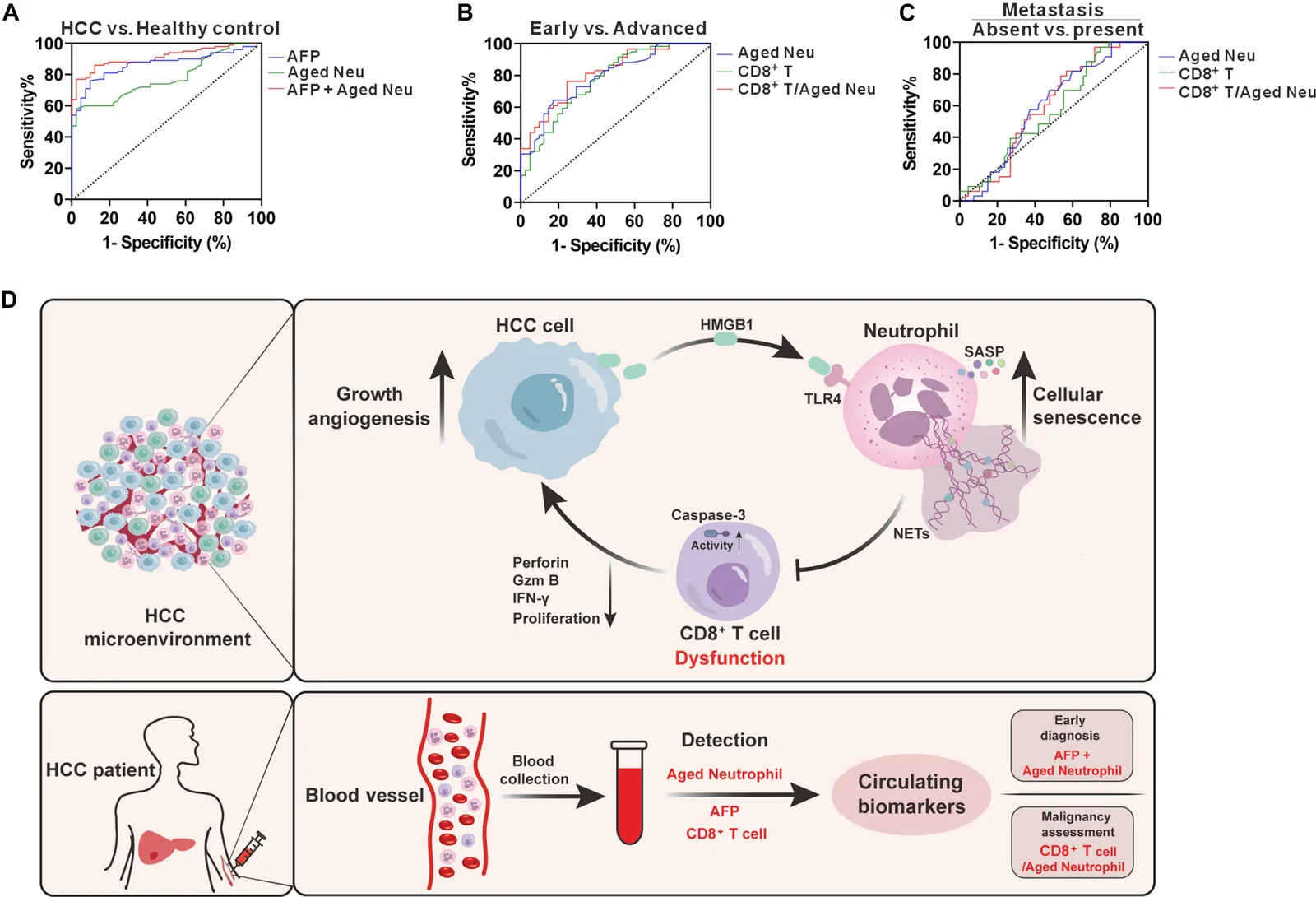
The clinical significance of detecting circulating aged neutrophils in HCC patients. (A) ROC curve of AFP, Aged Neu, and AFP + Aged Neu for distinguishing HCC from healthy controls. (B) ROC curve of Aged Neu, CD8^+^ T, and CD8^+^ T/Aged Neu levels for identifying advanced stage from early. (C) ROC curve of Aged Neu, CD8^+^ T, and CD8^+^ T/Aged Neu levels for predicting metastasis in HCC. (D) Proposed model illustrating that HCC-derived HMGB1 activates TLR4 signaling, inducing neutrophil senescence, which in turn promotes HCC growth and angiogenesis by impairing CD8^+^ T cell function. Moreover, combined detection of circulating aged neutrophils and AFP may serve as a valuable diagnostic biomarker for HCC, while the CD8^+^ T/Aged Neu ratio holds strong potential for HCC grading and malignancy evaluation. ROC, receiver operating characteristic; AFP, α-fetoprotein; HCC, hepatocellular carcinoma; Aged Neu, aged neutrophils; HMGB1, high mobility group box 1; TLR4, Toll-like receptor 4; SASP, senescence-associated secretory phenotype, NETs, neutrophil extracellular traps.

Given the elevated proportion of circulating aged neutrophils and their tumor-promoting effect through suppression of CD8^+^ T cell function, we examined the distribution of aged neutrophils, CD8^+^ T cells, and the CD8^+^ T cell/aged neutrophil ratio in HCC patients stratified by various clinicopathological features (Table 2). The results showed that the proportion of aged neutrophils was markedly elevated in advanced- versus early-stage HCC patients [tumor, node, metastasis staging system (TNM) III/IV versus I/II]. Given the elevated levels of aged neutrophils in HCC patients, we further evaluated their diagnostic potential in distinguishing HCC from healthy controls. ROC analysis revealed that the combination of α-fetoprotein (AFP) and aged neutrophil levels yielded an AUC of 0.91, which was markedly higher than that of AFP alone (AUC = 0.87) or aged neutrophils alone (AUC = 0.78) (Fig. 8A), indicating that the combined measurement substantially improved diagnostic performance. In addition, we performed ROC analysis to assess whether aged neutrophils could serve as potential biomarkers for HCC malignant risk assessment. The AUCs for distinguishing early- and advanced-stage HCC were 0.79 for aged neutrophils, 0.77 for CD8^+^ T cells, and 0.82 for the CD8^+^ T cell/aged neutrophil ratio (Fig. 8B). This highlights that the ratio may provide better discrimination of disease stage than either parameter alone. For predicting metastasis, the AUCs were 0.60 for aged neutrophils, 0.57 for CD8^+^ T cells, and 0.60 for the CD8^+^ T cell/aged neutrophil ratio (Fig. 8C), suggesting that none of these indicators had marked predictive value for HCC metastasis. These findings indicated that the combination of circulating aged neutrophils and AFP provided enhanced diagnostic value for HCC, whereas the CD8^+^ T cell/aged neutrophil ratio served as a more effective indicator for HCC malignancy assessment.

**Table 2.** Distribution of aged neutrophils in peripheral blood from HCC patients (*n* = 100) with various clinicopathological parameters. Aged Neu, CD8^+^ T, and CD8^+^ T/Aged Neu are presented as median (interquartile range).

Parameter	Total (cases)	Aged Neu (% of Neu)	*P* value	CD8^+^ T (cells/μl)	*P* value	CD8^+^ T-to-aged Neu ratio	*P* value
Gender							
Male	69	0.76 (0.73)	0.907	415 (264)	0.837	510.2 (957)	0.923
Female	31	0.83 (0.66)		422 (337)		481 (658.3)	
Age (years)							
<60	59	0.72 (0.71)	0.403	414 (302)	0.411	637.5 (1,060)	0.342
≥60	41	0.84 (0.72)		416 (250)		485.1 (774.4)	
Tumor number							
Solitary	57	0.76 (0.70)	0.594	419 (300)	0.434	549 (873)	0.909
Multiple	43	0.74 (0.71)		440 (263)		494.3 (992)	
HBV							
<LLOQ	38	0.87 (0.72)	0.182	402 (264)	0.875	712.9 (756.7)	0.402
≥LLOQ	62	0.70 (0.69)		420 (310)		483.2 (9,530.76)	
ALT (U/l)							
<50	50	0.84 (0.67)	0.345	401 (284)	0.250	473.6 (722)	0.214
≥50	50	0.73 (0.73)		439 (286)		696.3 (1,002.8)	
AST (U/l)							
<40	46	0.87 (0.67)	0.179	408 (282)	0.505	438.8 (704.4)	0.171
≥40	54	0.72 (0.71)		426 (312)		688.5 (937)	
AFP (μg/l)							
<400	39	0.75 (0.65)	0.971	373 (206)	0.067	465.8 (822.8)	0.430
≥400	61	0.76 (0.80)		439 (335)		549 (884.4)	
TNM stage							
I + II	41	0.54 (0.5)	<0.001	555 (324)	<0.001	971.4 (1,329.2)	<0.001
III + IV	59	0.96 (0.76)		364 (208)		371.2 (479.8)	
Metastasis							
Absent	67	0.70 (0.76)	0.125	419 (326)	0.227	646.6 (1,203.8)	0.114
Present	33	0.88 (0.57)		414 (248)		460.3 (496.1)	

Aged Neu, aged neutrophils; HCC, hepatocellular carcinoma; LLOQ, lower limit of quantification

## Discussion

This study focused on a pathologically altered neutrophil subset, aged neutrophils, with the aim of systematically characterizing their distribution and functional status within the HCC microenvironment and assessing their influence on CD8^+^ T cell activity. Our findings demonstrated that aged neutrophils were significantly enriched in the peripheral blood and tumor tissues of HCC patients, and they promoted tumor growth by inhibiting CD8^+^ T cell function through NET release. Further analysis demonstrated that combining AFP and aged neutrophils improved early diagnostic performance, and the CD8^+^ T cell/aged neutrophil ratio showed excellent discrimination between tumor stages, indicating its potential clinical value as a biomarker (Fig. 8D).

Neutrophils are the most abundant leukocytes in peripheral blood, but they have a short lifespan of less than 24 h under normal conditions [23]. Recent studies have highlighted their notable heterogeneity, especially within the TME, where neutrophils with distinct phenotypes exert opposing effects [29]. Tumor-associated neutrophils can be broadly categorized as anti-tumor N1 or pro-tumor N2 subtypes: N1 neutrophils show strong cytotoxicity, produce reactive oxygen species and pro-inflammatory factors, enhance T cell responses, and restrain tumor growth, whereas N2 neutrophils express immunosuppressive molecules such as arginase 1 (ARG1), IL-10, and transforming growth factor-β, promote angiogenesis, suppress CD8^+^ T cell activity, and facilitate tumor progression [5,30,31]. This functional complexity and plasticity have attracted considerable interest, and better understanding of their roles may help refine immunoregulatory strategies and improve anti-tumor therapies. After release from the bone marrow, neutrophils undergo changes in phenotype and function over time, a process known as neutrophil senescence [16,21,23,32]. While senescence helps maintain immune homeostasis, excessive accumulation under pathological conditions can lead to tissue damage and immune dysregulation [20]. Aged neutrophils up-regulate the chemokine receptor CXCR4 on their surface, promoting their migration back to the bone marrow in response to C–X–C motif chemokine ligand 12 (CXCL12), where they are cleared by macrophages; meanwhile, their expression of CD62L decreases [19]. High expression of integrin subunits CD11b and CD49d indicates that aged neutrophils may more readily adhere to inflamed endothelial cells [16]. Considering the important role of neutrophils in tumor progression, we further explored the aged neutrophils in HCC and found significant enrichment of aged phenotype (CXCR4^hi^CD62L^lo^) neutrophils in the peripheral blood and tumor tissues of HCC patients. Similar enrichment had been reported in inflammatory diseases [33], infections [23], cardiovascular conditions [34], and various cancers, including melanoma [35], breast cancer [21], and prostate cancer [22].

Previous investigations have predominantly characterized neutrophil function in their mature circulating form, but emerging evidence highlights functional heterogeneity at multiple stages of the neutrophil life cycle. Neutrophil senescence is regulated by both circadian rhythms and external stimuli [18,19]. Microbial metabolites could translocate across the gut barrier into circulation and promote neutrophil aging through TLR/myeloid differentiation primary response 88 signaling pathways [19]. In sepsis and sickle cell models, depletion of the gut microbiota significantly reduced the abundance of aged neutrophils and mitigated inflammatory damage, underscoring the crucial role of the microbiota in immune aging regulation [19]. In the context of cancer, tumor cells can accelerate neutrophil aging through diverse mechanisms. For instance, melanoma cells induced neutrophil senescence via angiotensin II [35], and breast cancer cells secreted nicotinamide phosphoribosyltransferase to activate sirtuin 1 signaling, driving neutrophils toward a CXCR4^+^ aged phenotype (CD45^+^CD11b^+^Ly6G^+^CXCR4^+^) [21], while in prostate cancer, the apolipoprotein E-enriched secretome activated the spleen tyrosine kinase pathway to promote neutrophil senescence [22]. Our study further demonstrated that HMGB1 derived from HCC cells drove neutrophil senescence via TLR4 signaling. HMGB1 is a nuclear nonhistone protein and a classic damage-associated molecular pattern (DAMP) molecule. It can be released—either actively or passively—during cellular stress, injury, or death, and acts as a potent pro-inflammatory mediator [36,37]. Recent findings point to a central role for HMGB1 in driving and upholding cellular senescence [38]. For example, HMGB1 released from the inflammatory bone marrow microenvironment promotes mesenchymal stem cell senescence through TLR2/4 and nuclear factor κB (NF-κB) pathways [28]. Pharmacological inhibition of HMGB1 with ethyl pyruvate had been shown to alleviate lupus nephritis and suppress the senescence-associated secretory phenotype [28,39]. Additionally, oxidized HMGB1 secreted by senescent fibroblasts triggered p53-dependent senescence via TLR4-mediated cytokine production [40]. Other DAMPs, such as S100A6, are also implicated in cellular senescence, as their knockdown induces G2/M arrest and senescence in endothelial and fibroblast cells [36]. Notably, different types of cancer appear to exploit distinct mechanisms to induce neutrophil senescence, reflecting the complexity and context-dependent regulation of this process in the TME.

Neutrophils support tumor progression through secretion of bioactive molecules, including neutrophil elastase (NE) for proliferation, matrix metalloproteinase 9 (MMP-9) and VEGF for angiogenesis, ARG1 for immune evasion, and NETs for metastasis [41]. Aged neutrophils exhibit elevated levels of NE, VEGF, MMP-9, ARG1, C–C motif chemokine ligand 3 (CCL3), CCL5, reactive oxygen species, and NETs compared to non-aged counterparts [23,32,35,41]. While senescence is necessary for immune regulation, excessive accumulation may lead to inflammation and dysfunction [20]. The extent of aging was associated with pro-inflammatory activity in neutrophils, and the number of aged neutrophils was associated with carotid atherosclerosis severity [19]. In cancer, aged neutrophils accumulated in the lungs and produced NETs to trap tumor cells, fostering pulmonary metastatic spread in breast cancer [21]. In immune regulation, aged neutrophils more strongly suppressed CD8^+^ T cell production of IFN-γ and TNF-α, with partial reversal by programmed cell death ligand 1 or ARG1 blockade, suggesting multiple mechanisms of immunosuppression [23]. In HCC patients, we observed a negative correlation between CXCR4^hi^CD62L^lo^ aged neutrophils and CD8^+^ T cells. CellChat analysis also indicated potential cell–cell interactions between aged neutrophils and CD8^+^ T cells. Mechanistically, aged neutrophils inhibited CD8^+^ T cell proliferation and expression of effector molecules (IFN-γ, Gzm B, perforin) through NETs. Our previous studies reported that NETs promote HCC growth, angiogenesis, and metastasis [9]. Previous studies had shown that NETs could suppress CD8^+^ T cell function by disrupting T cell receptor (TCR) signaling and inhibiting NF-κB nuclear translocation [42]. Accumulating evidence further indicated that distinct NET components exerted diverse biological effects via different signaling pathways. For instance, NET-derived DNA had been shown to bind coiled-coil domain containing 25 on the surface of tumor cells, promoting tumor migration and metastasis [43], while we previously reported that phagocytosed NET-DNA activated the absent in melanoma 2 inflammasome in macrophages, driving hepatic necroinflammatory injury and fibrosis [44]. Building on these findings, the present study demonstrated that NETs markedly induced caspase-3 activation in CD8^+^ T cells, and that enzymatic digestion of the DNA backbone within NETs significantly attenuated this effect, suggesting a DNA-dependent mechanism. Notably, similar phenomena had been reported in gastric cancer patients, where increased caspase-3 activity in T cells was closely associated with increased T cell apoptosis and reduced expression of the TCR ζ (CD3ζ) chain [45]. As a critical component of the TCR complex, CD3ζ is essential for antigen-induced T cell activation. Collectively, these findings suggested that enhanced caspase-3 activity might impair CD8^+^ T cell activation and effector function by disrupting TCR signaling integrity. Nevertheless, the specific NET components responsible for caspase-3 activation, along with the receptors and signaling pathways that mediate this effect in CD8^+^ T cells, are still unknown and require further systematic investigation.

Cytotoxic CD8^+^ T cells are central to antitumor immunity [13]. They release cytokines like TNF-α and IFN-γ to indirectly damage tumors, form immunological synapses to deliver perforin and granzymes that trigger apoptosis, and express FasL to induce Fas-mediated cell death [46–48]. CD8^+^ T cell function in the TME depends on effective antigen presentation, activation, trafficking, and differentiation [10]. However, the TME exerts immunosuppressive effects via nutrient depletion and immunosuppressive metabolites (adenosine, lactate), as well as checkpoint ligand expression that inhibit T cell responses [10,49]. Our in vivo experiments confirmed that aged neutrophil-induced CD8^+^ T cell dysfunction promoted HCC cell proliferation, inhibited apoptosis, and enhanced angiogenesis but did not affect migration/invasion. CD8^+^ T cells suppressed tumor angiogenesis primarily through IFN-γ, which inhibited endothelial cell proliferation and reduced VEGF-A while increasing CXCL9/10/11 to promote vessel normalization [50]. Aged neutrophil effects on HCC tumor growth were dependent on CD8^+^ T cell dysfunction. Interestingly, in lung metastasis models, although aged neutrophils impacted CD8^+^ T cells, they did not exert a measurable effect on metastasis, suggesting their role in modulating primary tumor immune surveillance rather than distant metastasis. Although numerous studies had indicated that reduced CD8^+^ T cell infiltration was frequently linked to high metastatic potential [51], other research had shown that depletion of CD8^+^ T cells alone did not significantly alter multi-organ metastasis in breast cancer [52]. These findings collectively suggested that CD8^+^ T cells are indispensable for effective immune surveillance in the primary tumor niche, while their influence on distant metastasis may be relatively limited or modulated by tumor-intrinsic factors and the surrounding microenvironment.

AFP remains the most widely utilized serum biomarker for HCC; however, its sensitivity is limited, particularly in early-stage or small tumors, and its levels can fluctuate with underlying liver disease [53]. AFP-L3%, although U.S. Food and Drug Administration (FDA)-approved for HCC risk stratification, suffers from low sensitivity, restricting its utility for disease monitoring. Similarly, PIVKA-II is employed for risk assessment but lacks FDA approval for surveillance purposes [53]. While circulating tumor cells offer direct evidence of malignancy, their scarcity and correlation with tumor burden hinder early detection [53]. Consequently, there remains a pressing need for novel circulating biomarkers. Given the elevated proportion, immunomodulatory functions, and accessibility of aged neutrophils in peripheral blood, we investigated their potential as biomarkers. Our findings demonstrated that combining AFP with aged neutrophil levels enhanced diagnostic accuracy for HCC, while the ratio of CD8^+^ T cells to aged neutrophils outperformed either marker alone in differentiating early from advanced disease stages. This study provided critical data supporting the development and clinical translation of novel biomarkers for HCC, offering promising strategies for earlier and more precise screening and staging in clinical practice. Such advances hold important potential to improve patient prognosis and facilitate personalized therapeutic decision-making, underscoring substantial academic significance and broad clinical applicability.

This study presents several limitations. Firstly, it remains unclear whether the alterations of enrichment and immunoregulatory functions of aged neutrophils are consistent across HCC of varying etiologies, including viral, alcoholic, and metabolic, and whether these findings extend to a broader pan-cancer context. Comprehensive systematic studies are needed to elucidate their roles across diverse tumor types. Specifically, different tumor types may employ distinct signaling pathways to drive neutrophil senescence. The specificity of the HMGB1–TLR4 axis derived from HCC in inducing neutrophil senescence, as proposed in this study, requires further verification. Given the limited ability of existing tumor cell lines to recapitulate the authentic TME, future studies using patient-derived organoids and primary HCC cells will be essential to substantiate the specificity and predominant role of the HMGB1–TLR4 axis in driving neutrophil senescence within the HCC microenvironment. Secondly, current in vitro methodologies for inducing aged neutrophils are limited and lack standardized protocols. Likewise, strategies for establishing in vivo models enriched with aged neutrophils require further refinement and diversification to strengthen the robustness of experimental conclusions. In addition, the precise mechanism by which NETs promote CD8^+^ T cell dysfunction via caspase-3 activation remains unclear, and the specific NET components as well as the TCRs responsible for caspase-3 activation in CD8^+^ T cells still need to be elucidated. Moreover, although our study preliminarily demonstrates the potential of combining serum AFP with aged neutrophil frequency for HCC diagnosis, and highlights the diagnostic utility of the CD8^+^ T cell to aged neutrophil ratio (CD8^+^ T/Aged Neu) for tumor staging, these biomarkers necessitate validation through prospective, multicenter, large-cohort studies encompassing diverse patient populations before clinical implementation can be realized.

## Conclusions

The present study demonstrates that HMGB1 derived from HCC induces neutrophil senescence via TLR4 signaling. These senescent neutrophils suppress CD8^+^ T cell function through NET release that activates caspase-3, thereby promoting HCC progression. Notably, combining serum AFP levels with the CXCR4^hi^CD62L^lo^ aged neutrophils provides superior diagnostic value for HCC compared with AFP alone. Moreover, the CD8^+^ T cell to senescent neutrophil ratio (CD8^+^ T/Aged Neu) emerges as a promising marker for identifying patients at high malignant risk. In summary, the results provide mechanistic insights into neutrophil senescence, identify potential immunotherapeutic targets, and propose valuable biomarkers for the diagnosis and clinical management of HCC.

## Ethics Approval and Consent to Participate

This study was approved by the Institutional Ethics Committee of the Second Affiliated Hospital of Chongqing Medical University in agreement with the Declaration of Helsinki (no. 2024-067). All procedures were performed in accordance with relevant ethical guidelines and regulations. Written informed consent for both participation in the study and publication of related data was obtained from all participants before inclusion. All animal procedures were approved by the Animal Ethics Committee of the Second Affiliated Hospital of Chongqing Medical University (approval no. IACUC-SAHCQMU-2024-00053).

## Data Availability

All data are included in the main text or Supplementary Materials. Additional data can be obtained from Liang Duan upon reasonable scientific request and completion of a material transfer agreement. The RNA-seq data reported in this study have been deposited in a publicly accessible repository under accession numbers GSE326268, GSE149614, and GSE189903 (https://www.ncbi.nlm.nih.gov/geo/).
