# Supplementary material for: Neutrophil Senescence Induced by Tumor-Derived HMGB1 Promotes Hepatocellular Carcinoma Progression through Neutrophil Extracellular Trap-Mediated Suppression of CD8+ T Cell Immunity
Source: Cancer Commun (Lond). 2026 Sep 11;46:0050. doi: 10.34133/cancomm.0050 (PMC13562795; doi:10.34133/cancomm.0050)
Supplement: Supplementary file 1 [file cancomm.0050.f1.zip › 25-00081 supplementary Figures - final.pdf]

Neutrophil senescence induced by tumor-derived HMGB1 promotes hepatocellular carcinoma progression through neutrophil extracellular trap-mediated suppression of $CD8^+$ T cell immunity

Yaqian Duan[1]†, Shan Yu[1]†, Xuanyi Wang[2]†, Rong Wu[1], Yuqing Zhao[1], Xuehua Kong[1], Shiyu Cao[1], Dan Xiang[1], Rui Wu[3], Jingying Zhou[4*], Yang Luo[5*], Liang Duan[1*]

1 Department of Laboratory Medicine, The Second Affiliated Hospital of Chongqing Medical University, Chongqing, P. R. China

2 Department of Pathology, The Second Affiliated Hospital of Chongqing Medical University, Chongqing, P. R. China

3 Department of Laboratory Medicine, The First Affiliated Hospital of Chongqing Medical University, Chongqing, P. R. China

4 School of Biomedical Sciences, The Chinese University of Hong Kong, Hong Kong SAR, P. R. China

5 Department of Laboratory Medicine, Chongqing General Hospital, School of Medicine, Chongqing University, Chongqing, P. R. China

†Yaqian Duan, Shan Yu and Xuanyi Wang contributed equally to this work.

[*] **Corresponding authors**:

Liang Duan, Department of Laboratory Medicine, The Second Affiliated Hospital of Chongqing Medical University, No.74 linjiang Road, Yu Zhong District, Chongqing, China.

Electronic address: **duanliang@cqmu.edu.cn**

Tel: + 86-023-63693193.

ORCID: https://orcid.org/0000-0002-3882-7527

Yang Luo, Department of Laboratory Medicine, Chongqing Center for Clinical Laboratory, Chongqing Academy of Medical Sciences, Chongqing General Hospital, School of Medicine, Chongqing University, No.118 Xingguang Avenue, Yubei District, Chongqing, 401147, China.

Electronic address: **luoy@cqu.edu.cn**

Tel: + 86-135-9408-8001.

ORCID: https://orcid.org/0000-0002-0166-9027

Jingying Zhou, School of Biomedical Sciences, The Chinese University of Hong Kong, Lo Kwee-Seong Integrated Biomedical Sciences Building, Area 39, Shatin, Hong Kong 999077, China.

Electronic address: **zhoujy@cuhk.edu.hk**

Tel: + 852 39431350.

ORCID: https://orcid.org/0000-0002-9740-6159

Supplementary Figures

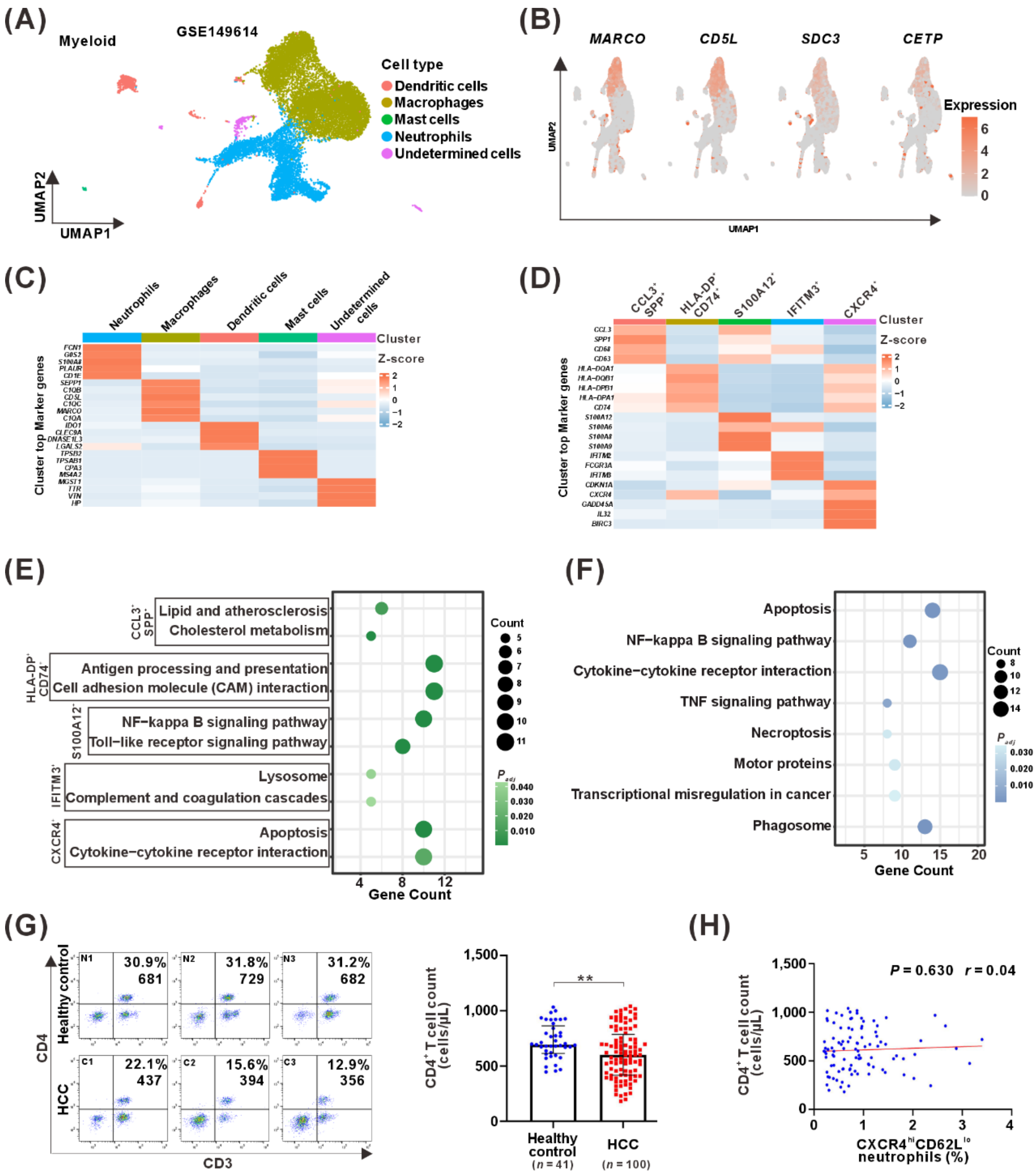

FIGURE S1. Altered abundance of aged neutrophils and their association with CD8^{+} T cells in HCC (Related to FIGURE 1). (A) UMAP of myeloid cells (GSE149614), annotated and colored by clustering. (B) UMAP color-coded for expression (gray to red) of key cell-type markers to define myeloid clusters. (C) Heatmap showing the top marker genes in each cluster. (D) Heatmap showing the top marker genes of each neutrophil subset. (E) KEGG pathway enrichment analysis for different neutrophil subsets. The bubble size corresponds to the number of involved genes, and the color gradient represents the significance level. (F) KEGG pathway

enrichment analysis for the $CXCR4^+$ neutrophil subset. The bubble size corresponds to the number of involved genes, and the color gradient represents the significance level. (G) Representative flow cytometry data and summary plot of the frequency showing $CD4^+$ T cell counts of Healthy control (n = 41) and HCC (n = 100). N1-N3 denote three independent healthy control samples, and C1-C3 denote three independent HCC samples. (H) Correlation between the percentage of $CXCR4^{hi}CD62L^{lo}$ neutrophil and $CD4^+$ T cell counts in HCC. The numbers shown below the percentage values indicate the absolute counts of $CD3^+CD4^+$ T cells per microliter of peripheral blood. **P < 0.010.

Abbreviations: UMAP; uniform manifold approximation and projection; KEGG, Kyoto Encyclopedia of Genes and Genomes; HCC, hepatocellular carcinoma; $CXCR4^{hi}CD62L^{lo}$, highly expressing CXCR4 and lowly expressing CD62L; P_{adj}, adjusted P value.

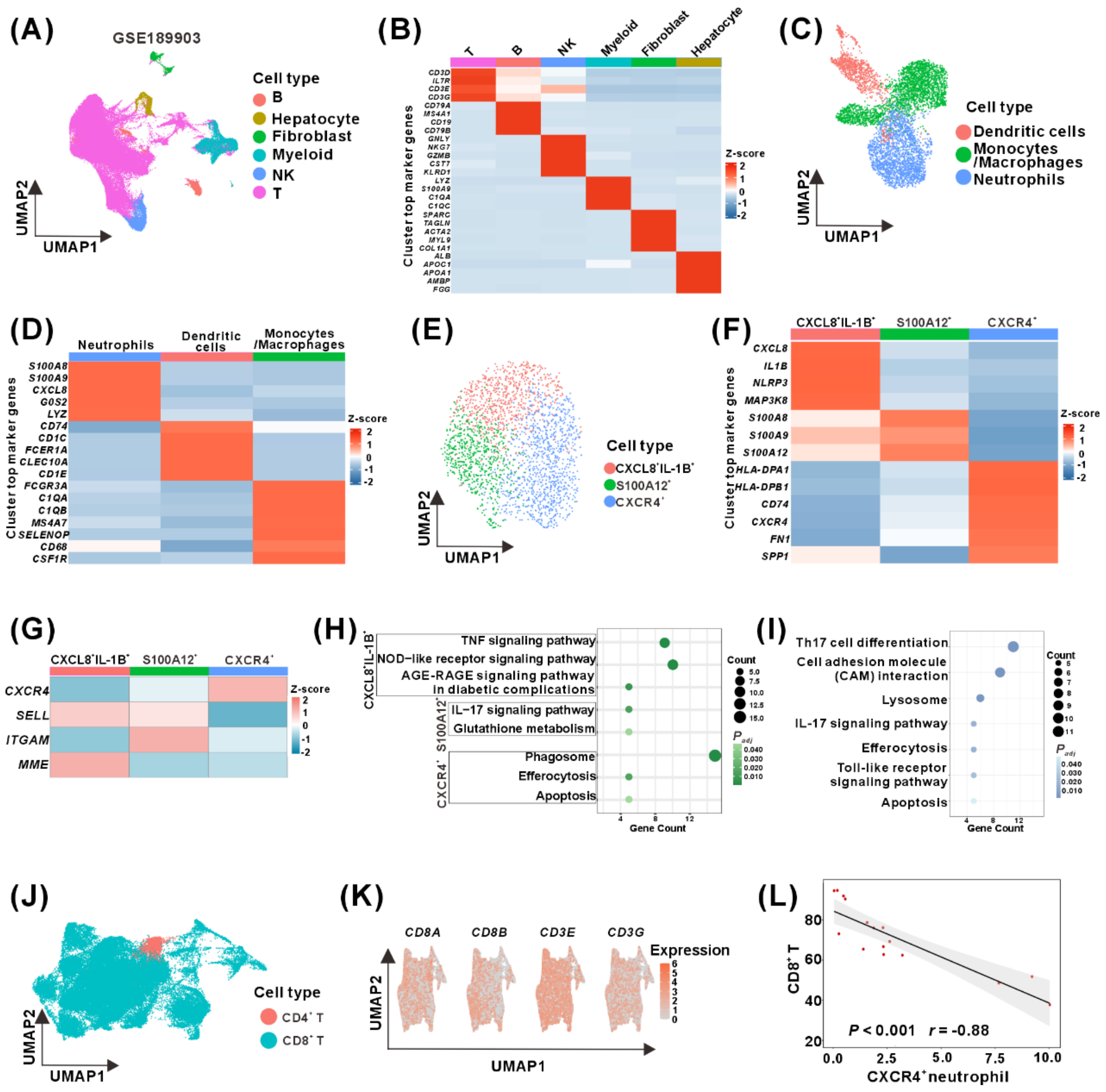

FIGURE S2. Altered abundance of aged neutrophils and their association with CD8^{+} T cells in HCC (Related to FIGURE 1). (A) UMAP of six cell clusters from the multicellular ecosystem of 16 HCC patients (GSE189903), colored by cell type. (B) Heatmap showing the top marker genes in each cluster. (C) UMAP of myeloid cells, annotated and colored by clustering. (D) Heatmap showing the top marker genes in each cluster. (E) UMAP of neutrophil subtypes, annotated and colored by clustering. (F) Heatmap showing the top marker genes of each neutrophil subset. (G) Heatmap showing the aged scores of different neutrophil subtype. (H) Bubble plot of KEGG pathway enrichment analysis for different neutrophil subsets. The bubble size corresponds to the number of involved genes, and the color gradient represents the significance level. (I) KEGG pathway enrichment analysis for the CXCR4^{+} neutrophil subset. The bubble size corresponds to the number of involved genes, and the color

gradient represents the significance level. (J) UMAP of T cell, annotated and colored by clustering. (K) UMAPs color-coded for expression (gray to red) of key cell-type markers to define $CD8^+$ T clusters. (L) Correlation between the abundance of $CXCR4^+$ neutrophils and $CD8^+$ T cells in the tumor microenvironment.

Abbreviations: UMAP; uniform manifold approximation and projection; HCC, hepatocellular carcinoma; NK, natural killer cell; KEGG, Kyoto Encyclopedia of Genes and Genomes; P_{adj}, adjusted P value.

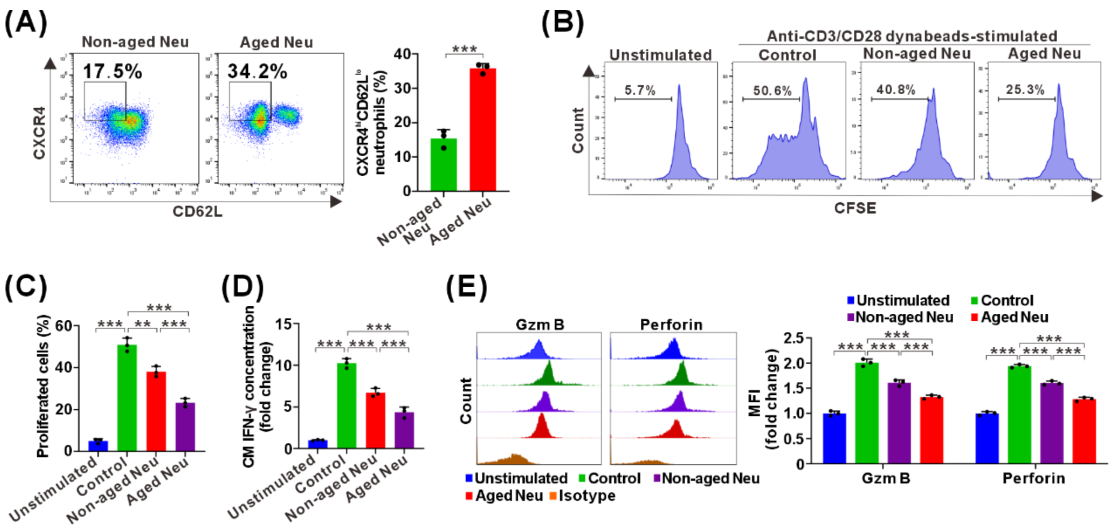

FIGURE S3. Effects of aged neutrophils on CD8^{+} T cell proliferation and activation (Related to FIGURE 2). (A) Representative FCM data and summary plot of the frequency showing the percentage of CXCR4^{hi}CD62L^{lo} neutrophils with (Aged Neu group) and without GM-CSF-treatment (Non-aged Neu group). (B-C) CFSE analysis of proliferative of CD8^{+} T cells co-cultured with Aged Neu or Non-aged Neu. (D) ELISA analysis for IFN-γ in CD8^{+} T cell-conditioned medium co-cultured with Aged Neu or Non-aged Neu. (E) Representative FCM data and MFI quantification of Gzm B and Perforin expression in CD8^{+} T cells co-cultured with Aged Neu or Non-aged Neu. $^{**}P < 0.010$, $^{***}P < 0.001$.

Abbreviations: FCM, flow cytometry; GM-CSF, Granulocyte–Macrophage Colony-Stimulating Factor; Non-aged Neu, non-aged neutrophils; Aged Neu, aged neutrophils; CXCR4^{hi}CD62L^{lo}, highly expressing CXCR4 and lowly expressing CD62L; CFSE, carboxyfluorescein succinimidyl ester; ELISA, enzyme-linked immunosorbent assay; CM, conditioned medium; MFI, mean fluorescence intensity; Gzm B, Granzyme B.

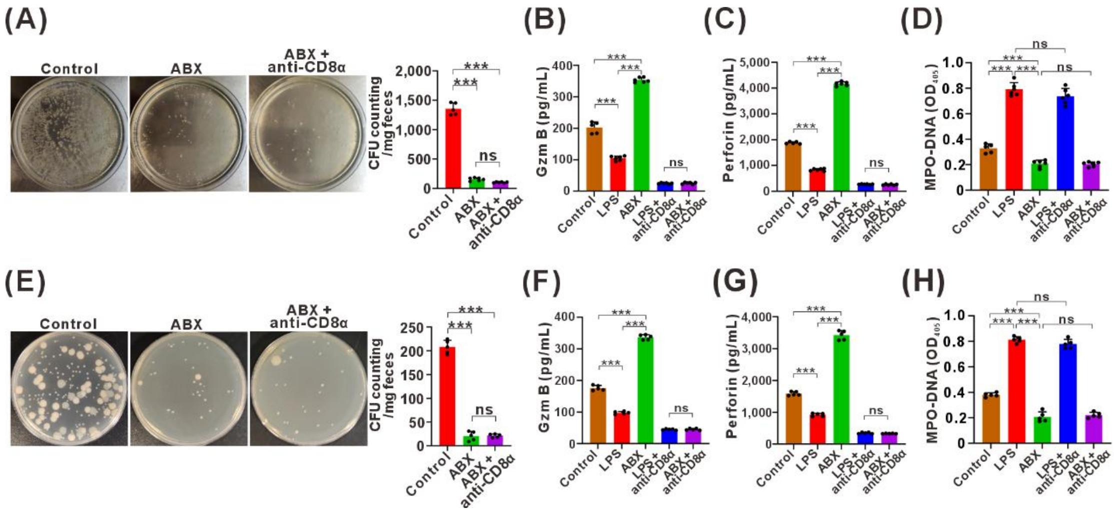

FIGURE S4. Aged neutrophils accelerated HCC progression via CD8^{+} T cell dysfunction *in vivo* (Related to FIGURE 5). (A) Representative images and quantification of fecal CFU counts in diethylnitrosamine and CCl_4-induced orthotopic HCC mice treated with or without ABX. (B-D) ELISA analysis serum for Gzm B, Perforin and MPO-DNA complexes in diethylnitrosamine and CCl_4-induced orthotopic HCC mice receiving different treatments: Control, LPS, ABX, LPS + anti-CD8α, and ABX + anti-CD8α. (E) Representative images and quantification of fecal CFU counts in syngeneic HCC transplantation mice treated with or without ABX. (F-H) ELISA analysis for serum Gzm B, Perforin and MPO-DNA complexes in syngeneic HCC transplantation mice receiving different treatments: Control, LPS, ABX, LPS + anti-CD8α, and ABX + anti-CD8α. ns, not significant; ***$P < 0.001$. Abbreviations: CCl_4, carbon tetrachloride; ABX, broad-spectrum antibiotic cocktail; CFU, colony-forming unit; ELISA, enzyme-linked immunosorbent assay; Gzm B, granzyme B; MPO, myeloperoxidase.

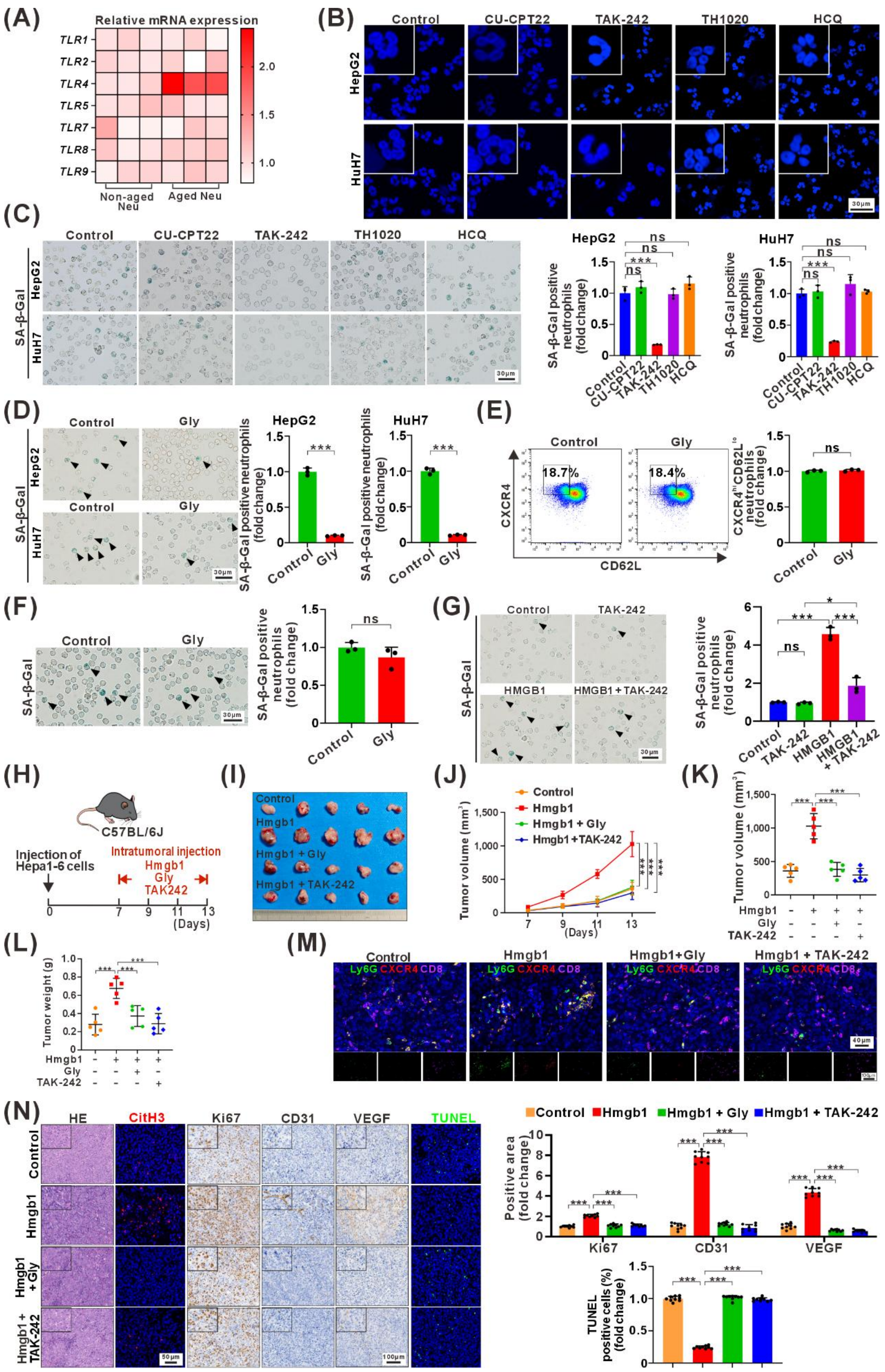

(A)
Relative mRNA expression
TLR1
TLR2
TLR4
TLR5
TLR7
TLR8
TLR9
Non-aged Neu
Aged Neu
(B)
Control
CU-CPT22
TAK-242
TH1020
HCQ
HepG2
HuH7
30μm
(C)
SA-β-Gal
HepG2
HuH7
SA-β-Gal positive neutrophils (fold change)
(D)
Control
Gly
SA-β-Gal positive neutrophils (fold change)
(E)
CXCR4
CD62L
18.7%
18.4%
neutrophils (fold change)
(F)
(G)
HMGB1
HMGB1 + TAK-242
(H)
C57BL/6J
Injection of Hepa1-6 cells
Intratumoral injection
Hmgb1
Gly
TAK242
(Days)
(I)
Control
Hmgb1
Hmgb1 + Gly
Hmgb1 + TAK-242
(J)
Tumor volume (mm³)
Hmgb1 + Gly
Hmgb1 + TAK-242
(K)
(L)
Tumor weight (g)
(M)
Hmgb1+Gly
Ly6G CXCR4 CD8
40μm
(N)
HE
CitH3
Ki67
CD31
VEGF
TUNEL
50μm
100μm
Positive area (fold change)
TUNEL positive cells (%) (fold change)

FIGURE S5. HMGB1 derived from HCC cells promoted neutrophil senescence via the TLR4 signaling axis (Related to FIGURE 7). (A) Heatmap showing the expression of *TLR1, TLR2, TLR4, TLR5, TLR7, TLR8* and *TLR9* in Non-aged Neu and Aged Neu by qRT-PCR analysis. (B) Representative fluorescence images of neutrophil nuclei (DAPI staining) following co-culture of neutrophil with HepG2 or HuH7 cells and treatment with CU-CPT22, TAK242, TH1020, or HCQ. (C) Representative images and quantification of SA-β-Gal staining following co-culture of neutrophil with HepG2 or HuH7 cells and treatment with CU-CPT22, TAK242, TH1020, or HCQ. (D) Representative images and quantification of SA-β-Gal staining following co-culture of neutrophil with HepG2 or HuH7 cells and treatment with Gly. The arrows indicate SA-β-Gal positive neutrophils. (E) Representative flow cytometry data and summary plot of the frequency showing the percentage of $CXCR4^{hi}CD62L^{lo}$ neutrophils following treatment with Gly. (F) Representative images and quantification of SA-β-Gal staining following treatment with Gly. The arrows indicate SA-β-Gal positive neutrophils. (G) Representative images and quantification of SA-β-Gal staining following treatment with HMGB1 or/and TAK242. The arrows indicate SA-β-Gal positive neutrophils. (H) Schematic diagram of the syngeneic HCC transplantation model established by subcutaneous injection of Hepa1-6 cells. Mice were subjected to different treatments: Control, Hmgb1, Hmgb1 + Gly, and Hmgb1 + TAK-242. (I) Images of bearing tumors from syngeneic HCC transplantation mice receiving different treatments: Control, Hmgb1, Hmgb1 + Gly, and Hmgb1 + TAK-242. (J) Tumor growth curve in syngeneic HCC transplantation mice receiving different treatments: Control, Hmgb1, Hmgb1 + Gly, and Hmgb1 + TAK-242. (K-L) Tumor volume and weight analysis in syngeneic HCC transplantation mice receiving different treatments: Control, Hmgb1, Hmgb1 + Gly, and Hmgb1 + TAK-242. (M) Representative images of IF staining for Ly6G, CXCR4 and CD8 in syngeneic tumor sections. (N) Representative images and quantification of HE staining, IHC staining for Ki67, CD31, VEGF, TUNEL staining and IF staining for CitH3 in syngeneic tumor sections. ns, not significant; *P < 0.050, ***P < 0.001.

Abbreviations: Non-aged Neu, non-aged neutrophils; Aged Neu, aged neutrophils; qRT-PCR; quantitative real-time PCR; DAPI, 4’, 6-diamidino-2-phenylindole; TLR, toll-like receptor; HCQ, hydroxychloroquine; SA-β-Gal, senescence-associated β-galactosidase; Gly, glycyrrhizic acid; $CXCR4^{hi}CD62L^{lo}$, highly expressing CXCR4 and lowly expressing CD62L; HMGB1, high mobility group box 1; Hmgb1, murine recombinant high-mobility group box 1 protein; IF, immunofluorescence; HE, hematoxylin and eosin; IHC, immunohistochemistry; VEGF, vascular endothelial growth factor; TUNEL, terminal deoxynucleotidyl transferase dUTP nick end labeling; CitH3, citrullinated modification of histone 3.
